# Variations in Ecosystem‐Scale Methane Fluxes Across a Boreal Mire Complex Assessed by a Network of Flux Towers

**DOI:** 10.1111/gcb.70223

**Published:** 2025-05-05

**Authors:** Koffi Dodji Noumonvi, Mats B. Nilsson, Joshua L. Ratcliffe, Mats G. Öquist, Natascha Kljun, Johan E. S. Fransson, Järvi Järveoja, Anders Lindroth, Gillian Simpson, Jacob Smeds, Matthias Peichl

**Affiliations:** ^1^ Department of Forest Ecology and Management Swedish University of Agricultural Sciences Umeå Sweden; ^2^ Unit for Field‐Based Forest Research Swedish University of Agricultural Sciences Vindeln Sweden; ^3^ Centre for Environmental and Climate Science, Lund University Lund Sweden; ^4^ Department of Forestry and Wood Technology Linnaeus University Växjö Sweden; ^5^ Biodiversity and Ecosystem Services in a Changing Climate, Lund University Lund Sweden

**Keywords:** climate change, eddy covariance, high latitude mires, landscape scale variations, mesoscale, methane emissions, northern peatlands, peat physical and chemical properties, peatland complex, spatio‐temporal control

## Abstract

High latitude mires are key ecosystems in the context of climate change since they store large amounts of carbon while constituting an important natural source of methane (CH_4_). However, while a growing number of studies have investigated methane fluxes (FCH_4_) at the plot‐ (~1 m^2^) and ecosystem‐scale (~0.1–0.5 km^2^) across the boreal biome, variations of FCH_4_ magnitudes and drivers at the mesoscale (i.e., 0.5–20 km^2^) of a mire complex are poorly understood. This study leveraged a network of four eddy‐covariance flux towers to explore the spatio‐temporal variations in ecosystem‐scale FCH_4_ across a boreal mire complex in northern Sweden over 3 years (2020–2022). We found a consistent hierarchy of drivers for the temporal variability in FCH_4_ across the mire complex, with gross primary production and soil temperature jointly emerging as primary controls, whereas water table depth had no independent effect. In contrast, peat physical and chemical properties, particularly bulk density and C:N ratio, were identified as significant baseline constraints for the spatial variations in FCH_4_ across the mire complex. Our observations further revealed that the 3‐year mean annual FCH_4_ across the mire complex ranged from 7 g C m^−2^ y^−1^ to 11 g C m^−2^ y^−1^, with a coefficient of variation of 16% that is similar to the variation observed among geographically distant mire systems and peatland types across the boreal biome. Thus, our findings highlight an additional source of uncertainty when scaling information from single‐site studies to the mire complex scale and beyond. Furthermore, they suggest an urgent need for peatland ecosystem models to resolve the mesoscale variations in FCH_4_ at the mire complex level to reduce uncertainties in the predictions of peatland carbon cycle‐climate feedbacks.

## Graphical abstract

This study used methane emission data from four flux towers across a mire complex located in northern Sweden, over a period of 3 years (2020–2022). We found that plant productivity and soil temperature were the primary controls of the temporal variations in methane emissions, while physical and chemical properties of the peat soil determined their spatial variability across the mire complex. The significant variation in methane emissions observed within this single mire complex highlights the importance of considering local differences when predicting how these critical carbon‐storing ecosystems will respond to climate change.

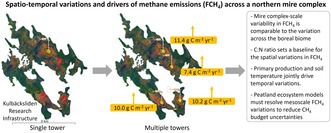

## 1 Introduction

Methane (CH_4_) is the most abundant trace gas in the atmosphere and a potent greenhouse gas that influences atmospheric temperature directly through its absorption of long‐wave radiation, and also indirectly by regulating ozone abundance in the troposphere and stratosphere through chemical reactions (IPCC 2023; Sobanaa et al. 2023; Wahlen 2003). High‐latitude mires (i.e., wetlands with active peat formation, Joosten and Clarke 2002) are an important natural source of CH_4_, emitting annually between 9 and 53 Tg CH_4_‐C (Peltola et al. 2019; Saunois et al. 2020; Yuan et al. 2024; Zhuang et al. 2006). The wide range and discrepancies between model‐based bottom‐up and atmospheric top‐down estimates, however, highlight a large uncertainty in CH_4_ emission rates (Zhu et al. 2025). The contribution of CH_4_ emissions from northern mires to the global CH_4_ budget requires particular attention as high‐latitude regions are undergoing the fastest changes in climate. These changes include rising temperatures, altered seasonality of precipitation (Sallinen et al. 2022) and water balance (Helbig et al. 2022), as well as longer growing seasons, all of which may exert direct feedbacks on CH_4_ emissions from northern mires (Tiwari et al. 2020). At present, however, our understanding of the interaction of peatland CH_4_ emissions with climate change is limited (Rosentreter et al. 2024).

The key challenge in estimating peatland CH_4_ fluxes (FCH_4_) is their considerable variation in response to small‐scale (1–10 m^2^) patterns in microtopography, which includes the distinct microforms of hummocks (always above the water table level), lawns (floating just above the water table level and occasionally flooded), hollows (temporarily flooded) and shallow pools (always flooded) (Granberg et al. 1997; Nilsson and Öquist 2009). Each of these microforms features unique biogeochemical and physical properties, which create strong gradients in the controls of CH_4_ production and consumption across a mire site. Studies using the chamber technique have provided valuable insights into the drivers and variability of FCH_4_ in dependence of microforms (e.g., Bubier et al. 1993; Perryman et al. 2022; Turetsky et al. 2014). For instance, higher CH_4_ production and lower CH_4_ oxidation are commonly observed in hollows compared to hummocks (Bubier et al. 1993; Perryman et al. 2022). However, scaling this spatial variability of FCH_4_ to larger mire areas based on plot‐scale chamber measurements has remained a major challenge.

Recent advances in analyser technologies have made it possible to overcome the spatial and temporal limitations of the chamber method and to quantify FCH_4_ at the ecosystem scale (Kljun et al. 2015; Vesala et al. 2008), using the eddy covariance (EC) technique. EC is a widely used method for direct and reliable measurements of gas and energy exchange between the atmosphere and ecosystems at high temporal resolution (i.e., half‐hourly) and all year‐round (Baldocchi 2014; Burba and Anderson 2010; Franz et al. 2018). More recently, the EC method has also become the state‐of‐the‐art approach for ecosystem‐scale FCH_4_ measurements in wetlands (Knox et al. 2019; Nemitz et al. 2018) and FCH_4_ estimates based on EC measurements have been compiled into global databases for northern peatlands (Knox et al. 2019; Peltola et al. 2019). Studies based on these databases have significantly advanced our understanding of peatland FCH_4_ drivers by highlighting the importance of both abiotic (i.e., temperature, water table depth, nutrient status) (Hanis et al. 2013; Knox et al. 2021; Lhosmot et al. 2023) and biotic (i.e., plant‐derived substrate and oxygen supply as well as direct transport of CH_4_ via arenchymatic plant tissue) (Girkin et al. 2020; Stewart et al. 2024; Yuan et al. 2024) controls. Still, the footprint of EC measurements usually only captures a distinct area of a peatland ecosystem (Rößger et al. 2019). Furthermore, given the extensive need for resources, EC measurements are commonly not replicated and insights from single EC sites are instead extrapolated to larger scales assuming similar FCH_4_ dynamics in the surrounding mire landscape, with little or no validation (Levy et al. 2020; Tuovinen et al. 2019).

In the boreal biome, mires often occur as conglomerates within a landscape, termed mire complexes. A mire complex is characterized by interconnected hydrology, and yet has hydromorphologically distinct features, with a potential for differing physical and chemical properties (Ehnvall, Ratcliffe, et al. 2023; Ivanov 1981; Pakarinen 1995). The surrounding upland areas play a critical role in controlling water and nutrient inflow, thus influencing ecosystem functioning. Generally, larger mires show greater variability in nutrient status as well as in the physical and chemical properties of peat (Ehnvall, Ågren, et al. 2023). At such large scales, the combination of environmental gradients and site‐specific hydrological and geological conditions may lead to new driver hierarchies, possibly causing higher‐level dynamics across the mire complex that could differ from those observed at the individual ecosystem level within the area. At present, it remains highly uncertain how well single site FCH_4_ estimates represent variations across the mire complex since information on its dynamics at the mesoscale (0.5–20 km^2^; Rydin and Jeglum 2013) is lacking.

In this study, we used 3 years of EC‐derived FCH_4_ data from four mire sites located within a typical boreal oligotrophic fen‐type mire complex (Noumonvi et al. 2023) with the aim to investigate the variations in FCH_4_ across a mire complex. The specific objectives were to (1) determine the spatio‐temporal variability of ecosystem‐scale FCH_4_ across a mire complex, and (2) investigate the key drivers regulating the variations in FCH_4_ dynamics across a mire complex.

## 2 Materials and Methods

### 2.1 Study Area

**FIGURE 1:**
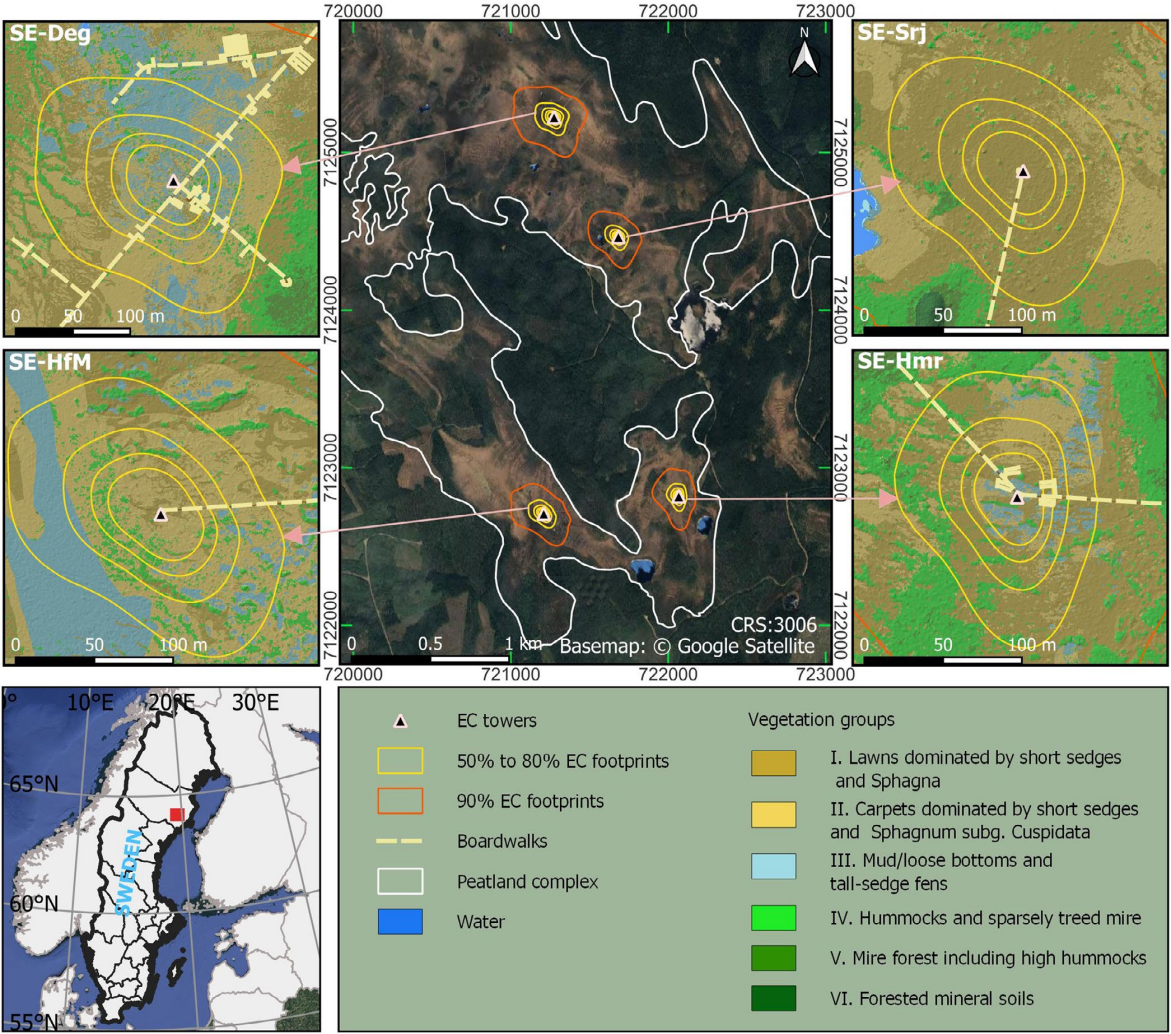
Study sites of the Kulbäcksliden Research Infrastructure (KRI) innorthern Sweden (lower left panel: Map lines delineate study areas and do not necessarily depict accepted national boundaries). The upper middle panel shows the KRI mire complex with a google satellite basemap. The four upper side panels provide close‐ups of the vegetation composition at the SE‐Deg, SE‐HfM, SE‐Hmr, and SE‐Srj sites, within the 50% to 80% footprint climatologies (May 2020–April 2021) calculated using the two‐dimensional Flux Footprint Prediction (FFP) model (Kljun et al. 2015) for the Eddy Covariance (EC) measurements. Details about the footprint calculation can be found in Section 2.3.

This study was conducted at the Kulbäcksliden Research Infrastructure (KRI), which includes four mire sites within a mire complex in northern Sweden: Degerö Stormyr (SE‐Deg), Hälsingfors Stormyran (SE‐HfM), Hålmyran (SE‐Hmr), and Stortjärn (SE‐Srj) (Figure 1) (Noumonvi et al. 2023). SE‐Deg is also part of the Integrated Carbon Observation System (ICOS; https://www.icos‐sweden.se/degero). The KRI is situated on an elevated land between two major rivers, Umeälven and Vindelälven, spanning latitudes 64°9′22.3″ N—64°11′22.7″ N, and longitudes 19°31′30″ E – 19°34′24.4″ E, about 10 km from the municipality of Vindeln (Figure 1).

The mire complex is a minerogenic and oligotrophic fen system situated on paragneiss bedrock, dating back to the Svecokarelian orogeny (1.92–1.87 billion years ago) (SGU 1963), resulting in a nutrient‐poor system (Ivarsson and Bjarnason 2009). Quaternary deposits consist of till‐based ridges of moraine. In many of the small depressions at the different mire catchments, peat deposits have accumulated, representing 46%–76% of each mire catchment, whereas histosols and podzols prevail in the upland catchment areas (Table 1).

**TABLE 1:** Site coordinates (i.e., EC tower locations) and catchment characteristics.

Variable	SE‐Deg	SE‐HfM	SE‐Hmr	SE‐Srj
Longitude (°E)	19.55654	19.55150	19.56924	19.56381
Latitude (°N)	64.18203	64.15956	64.16000	64.17498
Altitude (m a.s.l.)	266	292	290	269
Catchment area (ha)^a^	273	65	33	30
Mire/Catchment (%)	72	65	76	46
Dry bulk density 0–50 cm (kg/m^3^)^b^	75 ± 10	91 ± 6	81 ± 12	96 ± 9
C:N ratio 0–50 cm^b^	43 ± 3	39 ± 1	40 ± 3	34 ± 2

^a^ Catchments were delineated using hydrological flow analysis based on digital elevation models (Noumonvi et al. 2023). A catchment represents the land area from which water drains to a specific outlet point at each mire site.

^b^ Average ± standard error from three peat cores for dry bulk density and C:N ratio. The methodology for dry bulk density and C:N ratio calculation is described in Section 2.2.

The climate in the area is defined as subarctic (Dfc, cold temperate humid climate) according to the Köppen‐Geiger classification (Peel et al. 2007) with a long‐term (1991–2020) annual mean air temperature (Ta) and total precipitation of +3°C and 645 mm, respectively (based on data from the Kulbäcksliden SLU reference climate station). The average Ta for January and July is −7.2°C and + 15.4°C, while average total precipitation for January and July is 44 mm and 89 mm, respectively (Noumonvi et al. 2023).

The dominant vegetation communities based on the Finnish mire classification scheme (Eurola et al. 1995; Noumonvi et al. 2023) within the EC measurement footprints (Figure 1) can be categorized as follows:

- Lawns dominated by short sedges (*Eriophorum vaginatum, Trichophorum cespitosum*, *Carex pauciflora* , *Andromeda polifolia* , *Oxycoccus palustris*) and *Sphagna* ( *S. angustifolium* , *S. balticum* , *S. medium* , *S. rubellum* , *S. compactum* , *S. papillosum* );
- Carpets dominated by short sedges (same as in group I.), *Sphagnum* subg. *Cuspidata* ( *S. balticum* , *S. majus* , *S. lindbergii* , *S. jensenii* ), and *S. papillosum* or *S. compactum* ;
- Mud/loose bottoms and tall‐sedge fens ( *Scheuchzeria palustris* , *Carex limosa* , *Trichophorum cespitosum* , *Drosera longifolia* , *Sphagnum* subg. *Cuspidata*, *Cladopodiella fluitans* , *Menyanthes trifoliata* , *Carex rostrata* , *Sphagnum fallax* , *Warnstorfia* spp.);
- Hummocks and sparsely treed mires ( *Pinus sylvestris* , *Betula nana* , *Andromeda polifolia* , *Calluna vulgaris* , *Empetrum nigrum* , *E. hermaphroditum* , *Oxycoccus microcarpus*, *Vaccinium uliginosum* , *V. vitis‐idaea* , *Eriophorum vaginatum* , *Rubus chamaemorus* , *Sphagnum angustifolium* , *S. fuscum* , *S. medium* , *S. rubellum* , *Pleurozium schreberi* , *Cladonia mitis*, *C. stygia* ).

The areal contribution of each vegetation group (I–IV) varies among the four sites, but in general, groups II and III are the dominant ones at SE‐Deg and SE‐HfM, while drier lawn communities (group I) prevail at SE‐Hmr and SE‐Srj (Noumonvi et al. 2023).

### 2.2 Measurements of Ecosystem‐Scale CH_4_ Exchanges and Environmental Variables

The net ecosystem exchange of CO_2_ (NEE) and FCH_4_ was measured with EC systems. These included a 3D sonic anemometer (Gill HS‐50 at SE‐Deg, Metek uSonic‐3 Class A at the other sites), mounted on a boom, to measure the three components of wind velocity. Additionally, gas analyzers were employed (LI‐7200 and LGR FGGA 911–0010 at SE‐Deg, and a Picarro G2311‐f at the other sites, except for SE‐HfM, where an EC 155 and a LI‐7700 were temporarily used in 2020) for determining CO_2_, H_2_O, and CH_4_ concentrations. EC measurements were performed at a frequency of 20 Hz (LGR analyser) or 10 Hz (EC 155, LI‐7200, LI‐7700 and Picarro analyzers). The inlet of the tubes drawing air samples to the LI‐7200, LGR, and Picarro analyzers was close to the anemometer, with a vertical separation of less than 5 cm and northward separation of less than 18 cm. The tubes were 0.711, 20, and 6.9 m long, with diameters 5.3, 5.3, and 4.3 mm, and flow rates of 12, 12, and 5 L min^−1^ for the LI‐7200, LGR, and Picarro analyzers, respectively. The anemometers were set up at a height of 3.07 m at SE‐ Deg, and at a height of 2.75 m at the other sites. All sites were equipped with continuous mains power, and the sonic anemometers were heated during winter, making it possible to measure fluxes all year‐round.

The environmental variables measured at each mire site included Ta at 2 m height, soil temperature (Ts) at 2, 5 (only at SE‐Deg), 10, 15, 30, and 50 cm depth, water table depth (WTD), precipitation, in‐ and outgoing solar short‐ and terrestrial long‐wave radiation, and photosynthetically active radiation (PAR), with the respective instrumentations presented in Table S1. Ts and WTD measurements are replicated at five points at SE‐Deg and at two points at the other sites, mostly on lawns.

The normalized difference vegetation index (NDVI) is a widely used index for describing mire vegetation patterns and water table level at a landscape scale (Ehnvall, Ågren, et al. 2023; Kolari et al. 2021; Šimanauskienė et al. 2019). In this study, NDVI was derived from Sentinel‐2 multispectral instrument (MSI) images using the image collection ‘COPERNICUS/S2_SR_HARMONIZED’ on google earth engine (GEE 2024), which has a 10 m spatial resolution. Clouds and cloud shadows were masked out before aggregating the NDVI values over the 80% EC footprints of the four towers (see Section 2.3 for details on the EC footprint estimation). The nominal temporal resolution of Sentinel‐2 NDVI is 5 days, but over 30% of images were filtered out due to cloud pixels, yielding about 110 images for the 3 years of the study.

Based on vegetation inventory data from 20 plots at SE‐Deg and four plots at each of the three other sites (Noumonvi et al. 2023), above‐ground biomass (AGB) was estimated using a combination of leaf and stem counts, height measurements at the plots, and dry weights from samples collected outside the plots. This approach integrates non‐destructive methods, such as counting and height measurements, with destructive sampling for dry weight analysis, allowing for accurate biomass calculation per area unit following the ICOS protocol for quantifying mire vegetation (ICOS 2020). The AGB for sedges was also estimated by isolating the biomass associated with sedge species, that is, *Eriophorum* spp., *Tricophorum* spp., and *Carex* spp.

Peat cores (50 cm depth) were collected from lawns (representing 85% to 95% of the EC footprints, Figure S1) at each site in 2020. After extraction, the peat cores were frozen until further analysis. Bulk density as well as carbon (C) and nitrogen (N) content were determined across the 50 cm cores after drying them. C and N content were determined using a Thermo Fischer Scientific Flash EA 2000 elemental analyzer. These measurements were used to calculate the C:N ratio. Bulk density and C:N ratio per site were calculated by averaging data from the three cores for each site.

During the summer of 2022, samples for vertical peat soil CH_4_ gas profiles were collected. Peat pore space air (when sampled from above WTD) or water (when sampled from below WTD) was collected at two hollows and two hummocks per site, at different depths (5, 15, 25, 35, and 45 cm) and repeated four times (May, June, July, and August) over the sampling campaign. At SE‐Srj, and especially at lawns and depths below 25 cm, it was difficult to collect water samples due to the highly decomposed peat (reflected by the higher bulk density), leading to nine missing samples (out of the total planned 80 samples at this site) for the deeper peat. The pore gas/water samples were analysed for their concentration of CH_4_, using headspace gas chromatography. For sampling the air‐filled pore system above the ground water table, 5 mL of the pore gas phase was injected into 22 mL glass vials. For sampling the peat pore water in the saturated peat, 5 mL pore water was injected into 22 mL glass vials (with N_2_ at ambient pressure) containing 5 mL phosphoric acid (H_3_PO_4_, 85%), and conserved with a drop of ZnCl. CH_4_ and CO_2_ were analyzed by GC‐FID (PerkinElmer Clarus 580 equipped with a methanizer). Separation was carried out on an Elite‐PLOT Q column (30 m, 0.53 mm ID, 20 μm df, PerkinElmer) at 30°C with N_2_ as carrier gas (10 psi).

### 2.3 Eddy Covariance Data Processing

The EC data processing followed the best practices suggested by Nemitz et al. (2018), while adapting to more recent recommendations for some parts of the processing, such as gap‐filling (Irvin et al. 2021; Kämäräinen et al. 2022). High frequency mixing ratios or concentrations were processed using EddyPro flux calculation software, v.7.0.9 (LI‐COR Biosciences 2022), to produce half‐hourly CO_2_, CH_4_, and H_2_O fluxes. Time lags between measurements of wind velocity variables and gas concentrations were compensated using automatic time lag optimization with a narrow search window (generally less than 10 s) based on an initial assessment of the time lag. The processing also included tilt correction through a double rotation of the anemometer's axes (Wilczak et al. 2001), and the extraction of turbulent fluctuations from the high frequency time series using 30‐min block averaging. CH_4_ concentrations measured with the LGR analyser were first converted to mixing ratios (already available for all the other analyzers) before calculating fluxes, and therefore no Webb‐Pearman‐Leuning (WPL) correction was applied, except for the period of measurement with the open path analyser (Li‐7700) at SE‐HfM in 2020 (Table S1). Correction for spectral attenuation was performed according to Fratini et al. (2012).

NEE and FCH_4_ were quality‐checked and post‐processed in the R software v. 4.3.1 (R Core Team 2023), and all post‐processing performed was organised into the “PostEddyPro” R package v.0.1.0, available at https://github.com/bravemaster3/PostEddyPro. Specifically, a quality check of the EC data consisted of removing measurements that occurred at low signal strength of the EC instruments, and filtering out data collected in a non‐steady state or low turbulent conditions (Mauder and Foken 2004). Furthermore, fluxes measured under low friction velocity, that is, 0.1 m s^−1^ threshold determined following the method described by Reichstein et al. (2005), were removed. Percentages of original flux data left before gap‐filling ranged between 55% and 62% depending on the site, for all 3 years together (Figures S2–S4), and between 20% and 70% during the frozen seasons, and between 30% and 75% during the frost‐free seasons, depending on the site and year (Table S2). The frost‐free season was defined in this study as the period of the year where Ts at 10 cm depth remained consistently above 1°C for at least five consecutive days. The 10 cm depth for Ts was chosen based on the average WTD (~7 cm below the peat surface, varying between 5 cm and 10 cm across all sites during 2020–2022), since this depth is deemed most significant for the activity of methanogens and methanotrophs (Granberg et al. 1997). We refer to the period outside the frost‐free season as the frozen season. The sign convention adopted for NEE and FCH_4_ is positive for an emission of CO_2_ and CH_4_ by the ecosystem, and negative for CO_2_ and CH_4_ uptake by the ecosystem. Environmental variables were gap‐filled between sites, taking advantage of the availability of data at one site when data were missing at the other, using linear regression with the most relevant variables.

Gap‐filled environmental variables were used to train machine learning models to gap‐fill FCH_4_ and NEE. Random forests were used for FCH_4_ according to Irvin et al. (2021), while XGBoost was used to gap‐fill NEE according to Kämäräinen et al. (2022) and Vekuri et al. (2023). The coefficient of determination (*R* ^2^) of predicted vs. gap‐filled fluxes for holdout sets during the 10‐fold cross validations ranged between 0.88 and 0.95 for FCH_4_ and between 0.9 and 0.93 for NEE. Environmental variables used as predictors for gap‐filling FCH_4_ were Ta, Ts (10 cm), WTD, air pressure (Pa), incoming PAR (PARin), outgoing PAR (PARout), relative humidity (RH), vapour pressure deficit (VPD), and precipitation. Variables used for gap‐filling NEE were Ta, Ts, global radiation, VPD, and RH. In addition to the previous environmental variables, indicators of the time of the year such as yearly sine, yearly cosine, and time delta (Irvin et al. 2021) were also used as predictors for both FCH_4_ and NEE. Gap‐filled NEE fluxes were further partitioned into gross primary production (GPP) and ecosystem respiration (Reco), based on the nighttime partitioning approach implemented in the “ReddyProc” R package v. 1.3.2 (Wutzler et al. 2018). Both FCH_4_ and GPP used in further analyses were then aggregated to the relevant aggregation periods, that is, daily, frost‐free season, and annual sums. Flux uncertainties in terms of standard deviations were estimated through Monte Carlo simulation (Richardson and Hollinger 2007).

The integrated source area, or footprint climatology, for the flux measurements was determined using the two‐dimensional Flux Footprint Prediction (FFP) model (Kljun et al. 2015). The footprint climatology used in this study was computed using all available half‐hourly data from the period from May 2020 to April 2021, at intervals representing 50%, 60%, 70%, 80%, and 90% of cumulative source area coverage (Noumonvi et al. 2023). The model input parameters include the roughness length, measurement height above displacement height, friction velocity, Obukhov length, standard deviation of lateral wind speed, and boundary layer height. While most inputs were derived from EC data, boundary layer height was sourced from the ERA5 reanalysis dataset (Hersbach et al. 2020).

### 2.4 Global Fluxnet‐CH_4_ and Historic SE‐Deg FCH_4_ Data

To contextualize FCH_4_ data from our four KRI sites, FCH_4_ data were extracted from the Fluxnet‐CH_4_ database (Delwiche et al. 2021; Knox et al. 2019) for mire sites within the circumboreal region (Loidi et al. 2023). Fluxnet‐CH_4_ site details and data years included in this study are presented in Table 2. Although several additional sites measure FCH_4_ using EC, data availability is currently restricted to eight sites (three bogs and five fens) in the circumboreal region, including 2014–2018 data for our SE‐Deg site. To provide a long‐term reference for SE‐Deg, we also report the historic data for FCH_4_ (2019) and GPP (2014–2019), which were processed according to the procedure described in Section 2.3.

**TABLE 2:** Fluxnet FCH_4_ data included in the study.

Site ID	Country	Full name	Mire type	Years	Network	Latitude, longitude	Reference
SE‐Deg	Sweden	Degerö Stormyr	Fen	2014–2018	ICOS, Fluxnet	64.1820, 19.5565	Nilsson and Peichl (2020)
CA‐SCB	Canada	Scotty Creek Bog	Bog	2014–2017	Ameriflux	61.3089, −121.2984	Sonnentag and Helbig (2020)
FI‐Si2	Finland	Siikaneva‐2 Bog	Bog	2012–2016	Fluxnet	61.8372, 24.1967	Vesala, Tuittila, Mammarella, and Alekseychik (2020)
US‐BZB	USA	Bonanza Creek Thermokarst Bog	Bog	2014–2016	Ameriflux	64.6955, −148.3208	Euskirchen and Edgar (2020a)
FI‐Lom	Finland	Lompolojankka	Fen	2006–2010	ICOS, Fluxnet	67.99724, 24.209179	Lohila et al. (2020)
FI‐Sii	Finland	Siikaneva	Fen	2013–2018	ICOS, Fluxnet	61.83265, 24.19285	Vesala, Tuittila, Mammarella, and Rinne (2020)
SE‐St1	Sweden	Stordalen grassland	Fen	2012–2014	Fluxnet	68.3541, 19.0503	Jansen et al. (2020)
US‐BZF	USA	Bonanza Creek Rich Fen	Fen	2014–2016	AmeriFlux	64.7013, −148.3121	Euskirchen and Edgar (2020b)

### 2.5 Data Analysis

#### 2.5.1 Correlation Analysis

Linear correlation analysis was performed to examine the relationships between annual FCH_4_ and various site characteristics (i.e., bulk density, C:N ratio, and mire/catchment ratio) and vegetation metrics (i.e., NDVI, GPP, and AGB of sedges as well as the proportion of the different vegetation groups). Separate analyses were conducted for each individual year (2020, 2021 and 2022) as well as for the three‐year average values. Correlation coefficients were considered statistically significant when the *p*‐value was < 0.05. To identify correlations between site characteristics and vegetation metrics, Pearson's correlation coefficients were calculated between all pairs of variables.

#### 2.5.2 Wavelet Coherence Analysis

Originally developed for signal processing and time frequency analysis involving two time series (Weng and Lau 1994), wavelet coherence analysis has emerged as a remarkably valuable tool within ecological contexts to visually represent the degree of coherence between two time series in both time and frequency domains (Campeau et al. 2024; Cazelles et al. 2008; Cho and Chon 2006). Wavelet analysis provides non‐stationary insights from time series data and reveals hidden patterns that might otherwise remain elusive, due to its capacity to unlock multi‐temporal scale correlations and agreement between two time series (Cazelles et al. 2008). In this study, we took advantage of wavelet transform to analyse the coherence between FCH_4_ and each of the three potential explanatory variables, GPP, Ts, and WTD. GPP here is considered to represent a proxy both for fresh substrate availability for methanogenesis and for vegetation phenology. Ts and WTD were both considered for their critical control on mire plant development and the activity of methanogens and methanotrophs involved in the production and oxidation, hence in the emission of CH_4_ (Granberg et al. 1997; Knox et al. 2021; Moosavi and Crill 1997).

#### 2.5.3 Commonality Analysis

Commonality analysis (Newton and Spurrell 1967) is a method for partitioning *R* ^2^ values from multiple regression analysis into the proportion of variance in the dependent variable explained by each independent variable uniquely, and the proportion of variance explained by common effects of predictors (Seibold and McPhee 1979). In this study, commonality analysis was used to decompose the total explained variance of daily FCH_4_ into first (unique), second (common effects between combinations of two variables) and third (common effects of all three independent variables) order effects, and to derive the total main effects (i.e., the sum of unique, second and third order combined effects) for each explanatory variable, i.e., GPP, Ts, WTD, as described in Koebsch et al. (2020). The commonality analysis was performed with the R package “yhat” v.2.0.3 (Nimon et al. 2021).

#### 2.5.4 Path Analysis

Path analysis is a statistical method used to describe the directed dependencies among a set of variables, for example, through concurrent multiple linear regressions (Streiner 2005). In this study, we designed a path diagram that consisted of a simple model, where all variables were observed, that is, no latent variable constructs were included. The path analysis follows the commonality analysis and tests the effect sizes of GPP, Ts, and WTD, adding PARin in a first part of the model where GPP depends on both PARin and Ts. In a second part of the model, GPP, Ts, and WTD explain FCH_4_. These paths are motivated by the fact that GPP can be closely related to both plant phenology and substrate supply for methanogenesis, whereas Ts regulates the activity of methanogens and methanotrophs which in turn depend on the respective anoxic and oxic conditions below and above the water table level, crucial for methane production and oxidation (Lai 2009; Yuan et al. 2022). All variables included in this analysis were scaled to unit variance, so that the resulting effect sizes are independent of the order of magnitude of the different variables. The application of this approach makes it possible to compare the effect size of the different independent variables in explaining FCH_4_ across the four sites. The path analysis was performed with the R package “lavaan” v. 0.6–16.

#### 2.5.5 Comparison of FCH_4_ Between the KRI Sites and Other Boreal Mires

To compare variations in annual FCH_4_ across our four KRI fen sites with that observed at other sites (bogs and other fens separately, but also both together) within the circumboreal region, a Kruskal–Wallis test was conducted as a non‐parametric alternative to one‐way ANOVA, given the limited number of site‐years. The Kruskal–Wallis test was followed by post hoc pairwise comparisons using the Dunn test to identify groups that exhibited statistically significant differences (i.e., *p* < 0.05). To adjust for multiple comparisons, a Bonferroni correction (Srinivasan et al. 2013) was applied.

## 3 Results

### 3.1 Variations of Environmental Variables Across the Mire Complex

**FIGURE 2:**
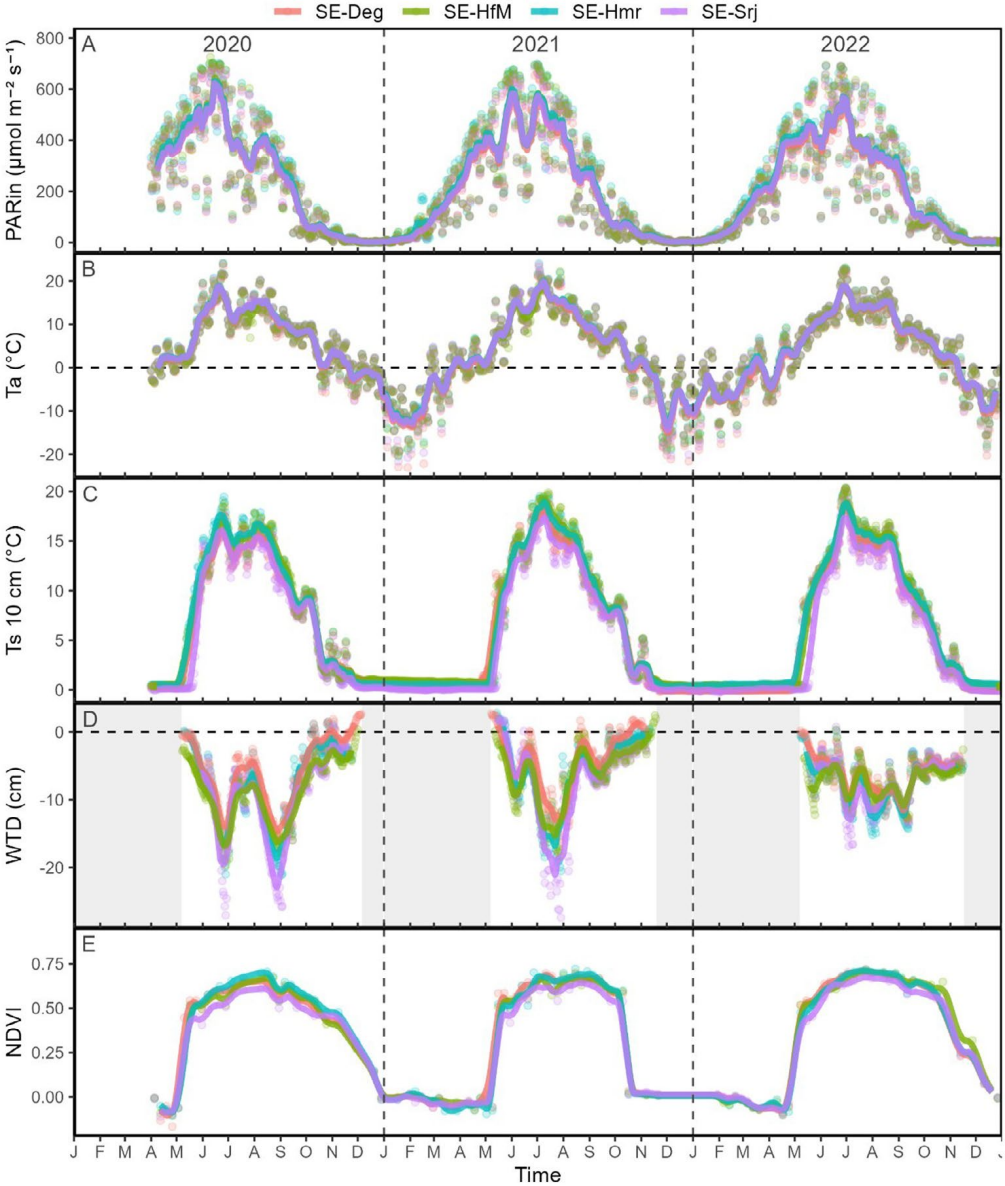
Daily means of environmental variables at the SE‐Deg, SE‐HfM, SE‐Hmr, and SE‐Srj sites during 2020–2022: (A) incoming photosynthetically active radiation (PARin), (B) air temperature at 2 m height (Ta), (C) soil temperature at 10 cm depth (Ts 10 cm), (D) water table depth (WTD), and (E) Sentinel‐2 derived NDVI. Solid lines represent the 15‐day running average. The shaded areas in panel “D” represent frozen seasons, that is, where Ts 10 cm is consistently below 1°C.

Given the spatial proximity of the four sites, meteorological factors such as Ta and PARin were very similar across the mire complex over the three study years (Figure 2A,B, Table 3), with average annual differences between sites less than 0.4°C and 25 μmol m^−2^, respectively, that is, within instrumental error. The soil environmental factors Ts and WTD showed some slight differences between sites (Figure 2C,D, Table 3). Specifically, the increase in Ts after snowmelt occurred 12 days later at SE‐Srj during 2020 and 2022 than observed at the other sites, although this difference was only observed at 10 cm depth (Figure S5). Still, the average annual difference in Ts at 10 cm depth between sites was generally less than 1°C, that is, within measurement error. The average frost‐free season WTD measured at lawns over 2020–2022, on the other hand, ranged from −5.2 cm at SE‐Deg and − 7.9 cm at SE‐Srj, that is, a maximum difference between sites of 2.7 cm. The daily minimum water table level was lower in 2020 at all sites compared to the subsequent years (< 1 cm lower than in 2021 and 4–10 cm lower than in 2022, depending on the site). During the peak growing season, the 3 years mean of NDVI was on average 9% lower (NDVI difference of ~0.05) at SE‐Srj compared to the other three mire sites (Figure 2E).

**TABLE 3:** Frost‐free season means (± standard deviation) of air temperature at 2 m height (Ta), soil temperature at 10 cm depth (Ts 10 cm), water table depth (WTD), incoming photosynthetically active radiation (PARin), and mean above‐ground biomass (AGB) at the SE‐Deg, SE‐HfM, SE‐Hmr, and SE‐Srj sites during 2020–2022.

Site	Frost‐free season^a^	Ta (°C)	Ts 10 cm (°C)	WTD (cm)	PARin (μmol m^−2^ s^−1^)	AGB (g m^−2^)
SE‐Deg	2020	8.3 ± 7.2	9.3 ± 5.3	−5.6 ± 5.3	268 ± 396	—
	2021	9.8 ± 6.7	11.1 ± 4.9	−4.1 ± 4.7	294 ± 400	—
	2022	9.6 ± 6.2	10.7 ± 5.1	−6.0 ± 3.1	277 ± 379	112 ± 9
SE‐HfM	2020	8.7 ± 7.1	10.2 ± 5.7	−8.6 ± 4.9	287 ± 410	—
	2021	9.3 ± 6.9	11.0 ± 5.8	−5.8 ± 4.8	291 ± 410	—
	2022	9.8 ± 6.3	11.1 ± 5.3	−7.1 ± 2.7	284 ± 390	89 ± 9
SE‐Hmr	2020	9.4 ± 6.6	10.6 ± 5.6	−7.6 ± 6	291 ± 421	—
	2021	10.0 ± 6.9	11.3 ± 5.5	−5.6 ± 5.5	300 ± 425	—
	2022	9.8 ± 6.3	11.0 ± 5.2	−7.0 ± 3.7	289 ± 404	101 ± 5
SE‐Srj	2020	10.0 ± 6.5	10.0 ± 5.2	−9.8 ± 6.6	275 ± 394	—
	2021	10.1 ± 6.8	10.4 ± 4.9	−7.0 ± 6.7	294 ± 403	—
	2022	8.9 ± 6.2	8.8 ± 5.3	−6.9 ± 3.2	230 ± 353	110 ± 7

^a^ Frost‐free season start and end dates are presented in Table S3.

### 3.2 Variations of FCH_4_ and GPP Across the Mire Complex

**FIGURE 3:**
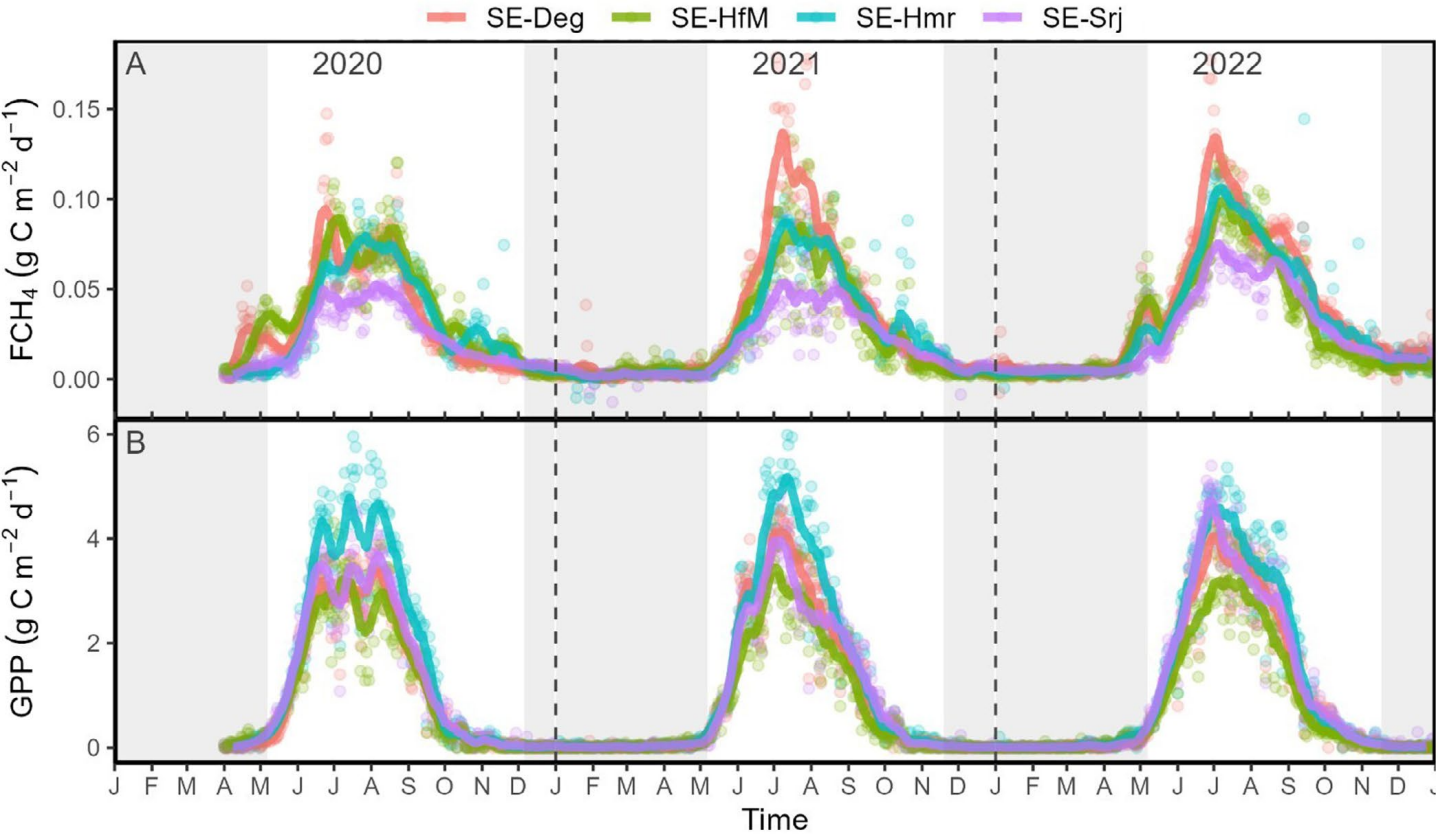
Daily sums of (A) methane fluxes (FCH_4_) and (B) gross primary production (GPP) at the SE‐Deg, SE‐HfM, SE‐Hmr, and SE‐Srj sites during 2020–2022. The shaded areas represent frozen seasons. Solid lines represent 15‐day running average.

Daily sums of FCH_4_ showed a 21% lower peak in 2020 compared to 2021–2022 at SE‐Deg, whereas a 27% higher peak in 2022 compared to 2020–2021 was noted at the other sites (Figure 3A). SE‐Srj exhibited the lowest FCH_4_ across all years, with the average peak being 40% lower than that of the other sites. Daily GPP sums were highest (4.9 g C m^−2^ d^−1^) and lowest (3.3 g C m^−2^ d^−1^) at SE‐Hmr and SE‐HfM, respectively, with limited inter‐annual variation (less than 5%) (Figure 3B). In comparison, peak daily GPP was 20% lower in 2020 relative to 2021–2022 at SE‐Deg and SE‐Srj. Maximum differences between sites in frost‐free season sums of FCH_4_ and GPP over the period 2020–2022 were on average 3 g C m^−2^ and 131 g C m^−2^, respectively (Table 4). A release of CH_4_ was observed in spring after snowmelt at SE‐Deg and SE‐HfM both in 2020 and 2022, and at SE‐Hmr only in 2022, whereas this early spring peak in CH_4_ release was not observed at SE‐Srj (Figure 3).

**TABLE 4:** Frost‐free season and annual sums (± standard deviation) of FCH_4_ and GPP at the four mire sites. Standard deviations were calculated using a Monte Carlo approach.

Site	Year	Frost‐free season		Annual	
		FCH_4_	GPP	FCH_4_	GPP
		(g C m^−2^)	(g C m^−2^)	(g C m^−2^)	(g C m^−2^)
SE‐Deg	2014	10.1 ± 0.03	292 ± 6	13.7 ± 0.03	304 ± 5
	2015	9.4 ± 0.02	277 ± 6	11.2 ± 0.03	278 ± 5
	2016	9.4 ± 0.02	206 ± 3	11.2 ± 0.03	207 ± 3
	2017	9.3 ± 0.03	221 ± 4	10.8 ± 0.03	222 ± 3
	2018	7.8 ± 0.02	116 ± 1	9.4 ± 0.02	117 ± 1
	2019	8.8 ± 0.05	272 ± 6	10.2 ± 0.06	298 ± 5
	2020	8.0 ± 0.04	317 ± 5	9.3 ± 0.04	320 ± 5
	2021	10.2 ± 0.06	342 ± 8	11.0 ± 0.06	344 ± 7
	2022	12.2 ± 0.06	362 ± 4	13.9 ± 0.06	366 ± 4
SE‐HfM	2020^a^	9.8 ± 0.07	295 ± 14	11.0 ± 0.07	298 ± 21
	2021	7.7 ± 0.03	275 ± 6	8.4 ± 0.03	277 ± 6
	2022	9.2 ± 0.03	290 ± 6	10.7 ± 0.03	296 ± 5
SE‐Hmr	2020^a^	8.3 ± 0.03	431 ± 7	9.4 ± 0.03	435 ± 7
	2021	8.3 ± 0.03	405 ± 8	9.0 ± 0.03	406 ± 8
	2022	10.8 ± 0.03	418 ± 7	12.1 ± 0.04	424 ± 7
SE‐Srj	2020^a^	5.6 ± 0.02	351 ± 6	6.4 ± 0.02	354 ± 6
	2021	5.7 ± 0.02	330 ± 7	6.5 ± 0.02	333 ± 7
	2022	7.9 ± 0.03	387 ± 8	9.4 ± 0.03	392 ± 9

^a^ Annual fluxes for 2020 at SE‐HfM, SE‐Hmr, and SE‐Srj were estimated from a linear relationship between the frost‐free season fluxes and annual fluxes from all sites together (Figure S6) because measurements started in April 2020.

The frost‐free season contributed on average between ~90% and ~99% to the annual FCH_4_ and GPP, respectively (Table 4). Historic data (2014–2019) from SE‐Deg reveals that the annual and frost‐free season FCH_4_ and GPP were mostly in range with more recent data (2020–2022), although GPP was ~50% lower in the drought year 2018.

### 3.3 CH_4_ Concentrations in Pore Air and Water Across the Peat Profile

**FIGURE 4:**
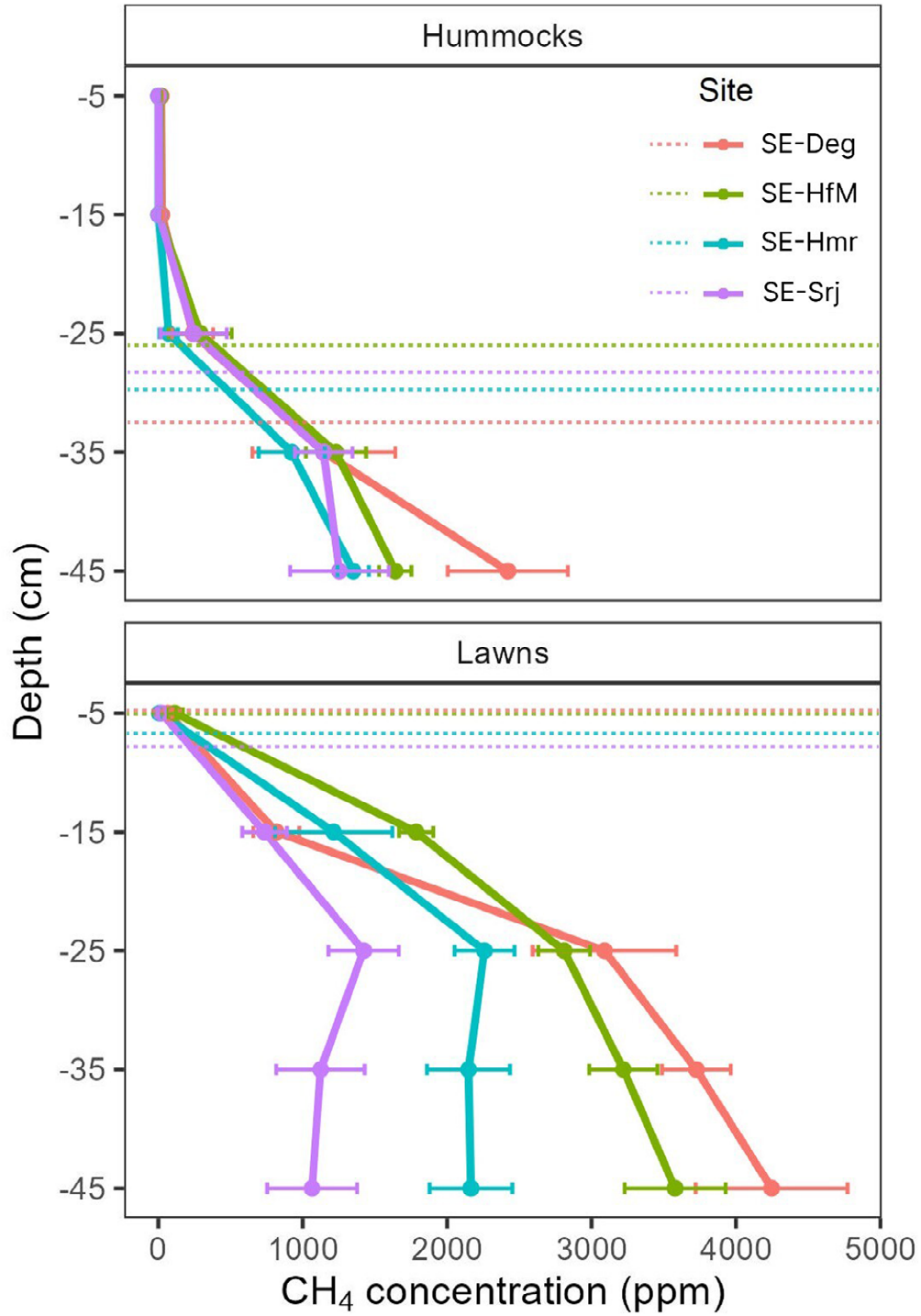
Methane (CH_4_) concentration (ppm) in the peat pore air (above WTD) or water (below WTD) at different depths at the SE‐Deg, SE‐HfM, SE‐Hmr, and SE‐Srj sites, split by microform. Hummock (top panel) and lawn (bottom panel) data are averages from two locations per site‐microform. The solid lines connect the average concentrations (dot markers) across all temporal replicates (end of May, June, July, and August 2022) per site‐microform‐depth, with the horizontal bars showing their standard errors. The dotted horizontal lines represent the average water table depth at each site‐microform across the temporal replicates.

The WTD at the sampled hummocks was on average between −25 cm and −35 cm for all sites, while that of lawns was closer to the surface, that is, at around −5 cm depth, during the year 2022. CH_4_ concentrations in pore air and water samples were relatively similar at hummocks across the four sites, ranging from an average (± standard error) of 470 ppm (± 281 ppm) at SE‐Hmr to 770 ppm (± 462 ppm) at SE‐Deg across all depths. In contrast, CH_4_ concentrations at lawns showed greater variability, with averages ranging from 873 ppm (± 239 ppm) at SE‐Srj to 2389 ppm (± 824 ppm) at SE‐Deg across all depths (Figure 4).

### 3.4 Drivers of Variations in FCH_4_ Across the Mire Complex

#### 3.4.1 Site Characteristics

**FIGURE 5:**
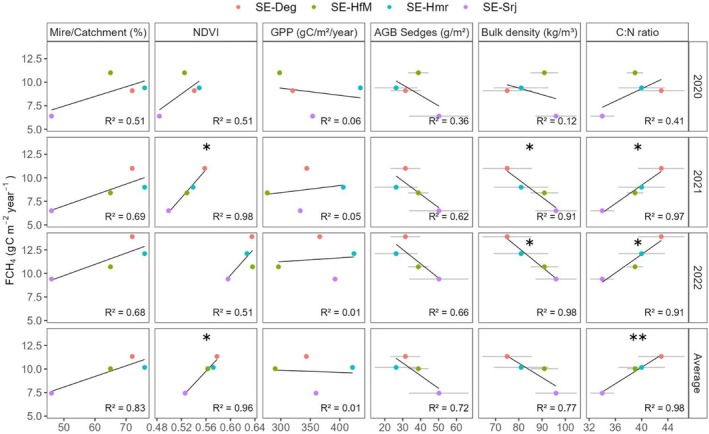
Correlation between annual FCH_4_ (each year, and averaged for all years) with site characteristics, that is, bulk density and C:N ratio at 0–50 cm depth and mire/catchment ratio, and vegetation metrics, that is, the normalized difference vegetation index (NDVI), gross primary production (GPP) and above‐ground biomass (AGB) of sedges. The grey horizontal bars represent the standard errors for the variables with spatial replicates. The ‘*’ represents *p*‐values < 0.05 and ‘**’ represents *p*‐values < 0.01, that is, the significance levels of the linear relationships. Panels without ‘*’ mean no significant linear relationship.

Annual sums of FCH_4_ showed a significant linear correlation with bulk density (negative, *p* < 0.05) and C:N ratio (positive, *p* < 0.05) across the four mire sites (Figure 5) except for year 2020, which was a drier year. The mire/catchment ratio exhibited a positive trend with FCH_4_, but there was no significant linear relationship. Averaged over all 3 years, FCH_4_ only showed a significant positive linear relationship (*p* < 0.01) with C:N ratio, while relationships with other site characteristics were non‐significant. The percentage of vegetation classes within the 80% EC footprint (Figure 1) did not significantly correlate with annual FCH_4_ (Figure S8), nor did sedge AGB or GPP (Figure 5). However, NDVI exhibited a significant positive correlation with annual FCH_4_ in 2021 and when averaged across the 3 years (Figure 5). Furthermore, higher sedge AGB seemed to be associated with lower FCH_4_, whereas GPP showed no clear trend with CH_4_ (Figure 5). It is further noteworthy that NDVI was not correlated with GPP and AGB, but instead was positively correlated with both mire/catchment ratio and with C:N ratio (Figure S9).

#### 3.4.2 Multi‐Temporal Coherence Between FCH_4_ and Environmental Variables

**FIGURE 6:**
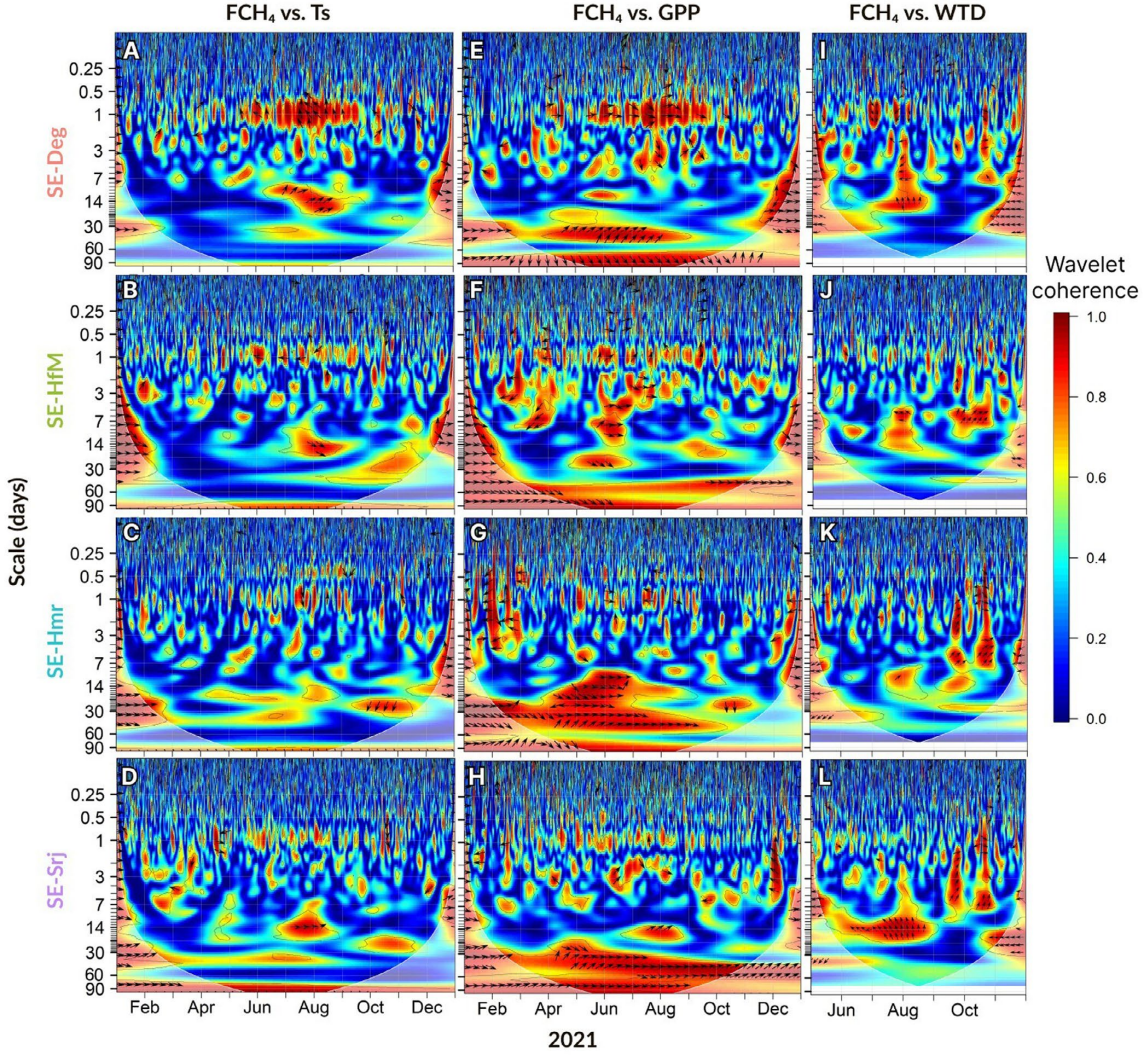
Wavelet coherence between half‐hourly methane fluxes (FCH_4_) and soil temperature at 10 cm depth (Ts) (A–D), gross primary production (GPP) (E–H), and water table depth (WTD) (I–L) in 2021, with each row representing one site (SE‐Deg, SE‐HfM, SE‐Hmr, SE‐Srj). Shaded areas at the bottom‐right and bottom‐left of each panel indicate areas outside the cone of influence, that is, impacted by edge effects. Arrows indicate the phase relationship between the two variables during high coherence periods (in red): In‐phase (rightward), in anti‐phase (leftward), variable x leading y (upward), or lagging y (downward) (Grinsted et al. 2004). Note the different *x*‐axis for panels I–L, where WTD is limited to the frost‐free season.

The coherence between the environmental variables and FCH_4_ in a time‐frequency domain is presented as wavelet coherence plots for the year 2021 (Figure 6), which generally reflect the dynamics of these relationships over the other years of the study (2020 and 2022, Figures S10 and S12). A significant and consistent coherence between FCH_4_ and Ts was observed at the daily timescale at all four mire sites throughout the frost‐free season (Figure 6A–D). This observation was particularly strong at SE‐Deg (Figure 6A) where the coherence between FCH_4_ and Ts persisted from June to September. However, the lead/lag phase relations between FCH_4_ and Ts were not consistent, as denoted by the different directions of the arrows over the frost‐free season, particularly at the daily timescale. During peak summer, that is, July, a synchronization between FCH_4_ and Ts appeared on bi‐weekly and tri‐weekly timescales, particularly at SE‐Deg (Figure 6A), SE‐HfM (Figure 6B), and SE‐Srj (Figure 6D).

Similarly to the coherence between FCH_4_ and Ts, there was a strong coherence between FCH_4_ and GPP from daily to 5‐day scale, especially at SE‐Deg (Figure 6E) and SE‐HfM (Figure 6F) in the summer (June–July–August). At the daily scale, FCH_4_ and GPP appeared to be in phase at SE‐Deg (Figure 6E), whereas FCH_4_ lagged ~4–6 h behind GPP at SE‐HfM (Figure 6F) during the summer. Strong multi‐weekly (3–8 weeks) coherences existed between FCH_4_ and GPP also in early spring and late autumn, especially at SE‐Hmr (Figure 6G) and SE‐Srj (Figure 6H).

In contrast to Ts and GPP (Figure 6A–H), there was no strong coherence between FCH_4_ and WTD at the daily timescale and at any of the sites (Figure 6I–L). Instead, high coherence events spanning from multi‐days to bi‐weekly timescales were noted in the middle of the summer (e.g., at SE‐Deg, Figure 6I) and in autumn (e.g., SE‐Hmr, Figure 6K and SE‐Srj, Figure 6L).

#### 3.4.3 Relative Importance of the Different Variables in Explaining Variations in FCH_4_

**FIGURE 7:**
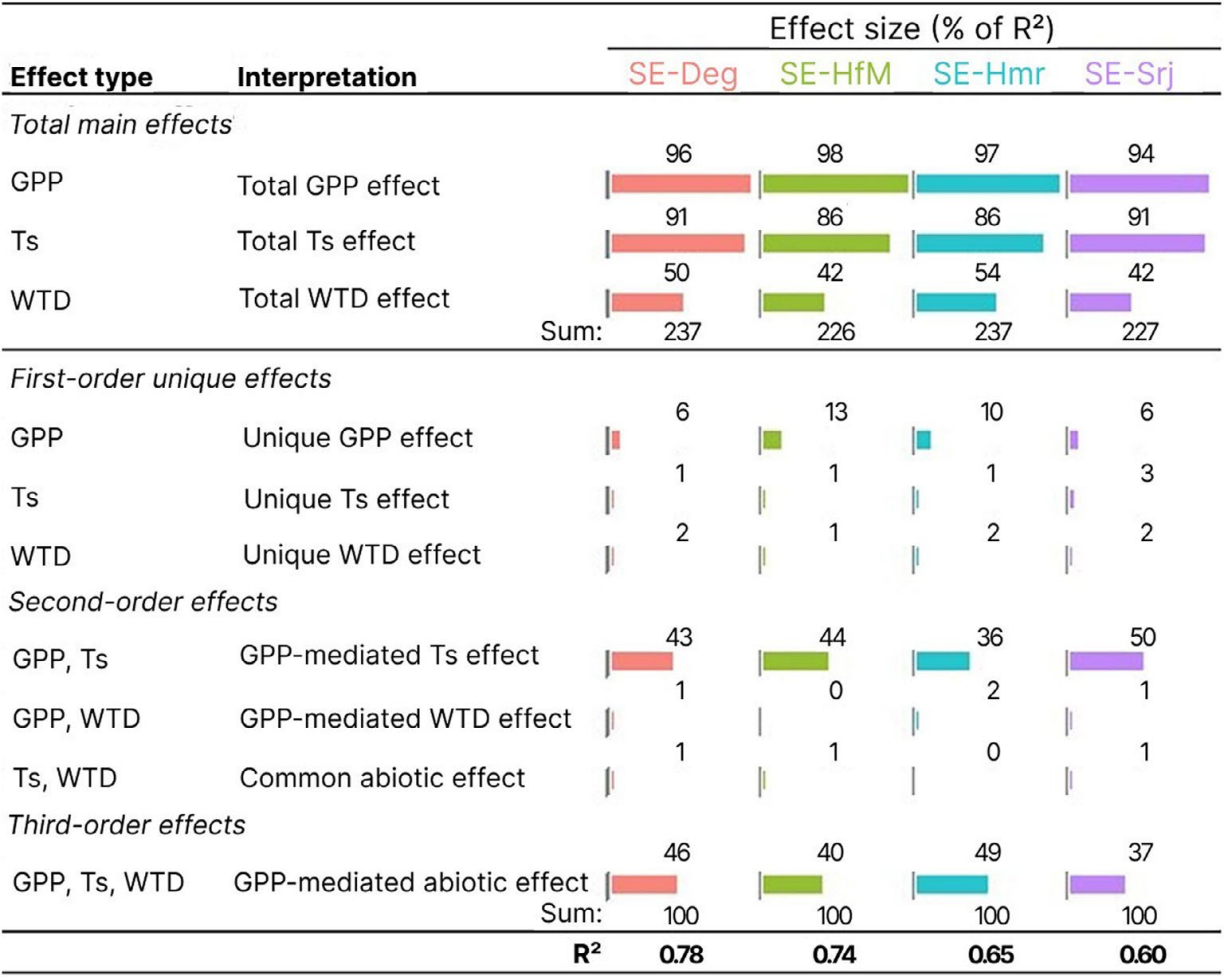
Commonality coefficients, that is, the percentage of *R* ^2^ explained by different independent variables or combinations of variables. This includes first‐order unique effects of gross primary production (GPP), soil temperature (Ts), and water table depth (WTD), second‐order effects of GPP‐Ts, GPP‐WTD, and Ts‐WTD; third‐order effects of GPP‐Ts‐WTD, as well as total main effects (i.e., defined as the sum of unique, second and third order combined effects for each variable) on daily FCH_4_ sum. Note that first, second, and third‐order effects sum up to 100% whereas total main effects exceed 100% due to collinearity. *R* ^2^ represents the total variance explained at each site.

The explained variance (*R* ^2^) in daily frost‐free season FCH_4_ as predicted by GPP, Ts, and WTD ranged from 0.60–0.78 depending on the site (Figure 7). The partitioning of the explained variance between the different explanatory variables through commonality analysis revealed weak first‐order unique effects of Ts and WTD (< 3% of *R* ^2^), and GPP (6%–13%). In comparison, several significant second‐order and third‐order common effects were noted. Specifically, important second‐order GPP‐mediated Ts effects were observed (43–50% of *R* ^2^ explained), while GPP‐mediated WTD effects and combined Ts and WTD effects were minimal (< 2% *R* ^2^ explained). Notably, third‐order effects, that is, GPP‐mediated abiotic (joint Ts and WTD) effects, contributed 37%–49% to the explained variance in FCH_4_. Across all sites, GPP and Ts showed higher total effects in explaining FCH_4_ compared to WTD, with the lowest WTD effects observed at both SE‐HfM and SE‐Srj. The key distinction among sites lies in the values of *R* ^2^, with wetter sites (SE‐Deg and SE‐HfM) exhibiting a higher value (0.78 and 0.74) and drier sites (SE‐Hmr and SE‐Srj) showing a lower value (0.65 and 0.60) of total percentage of explained variance in daily FCH_4_.

**FIGURE 8:**
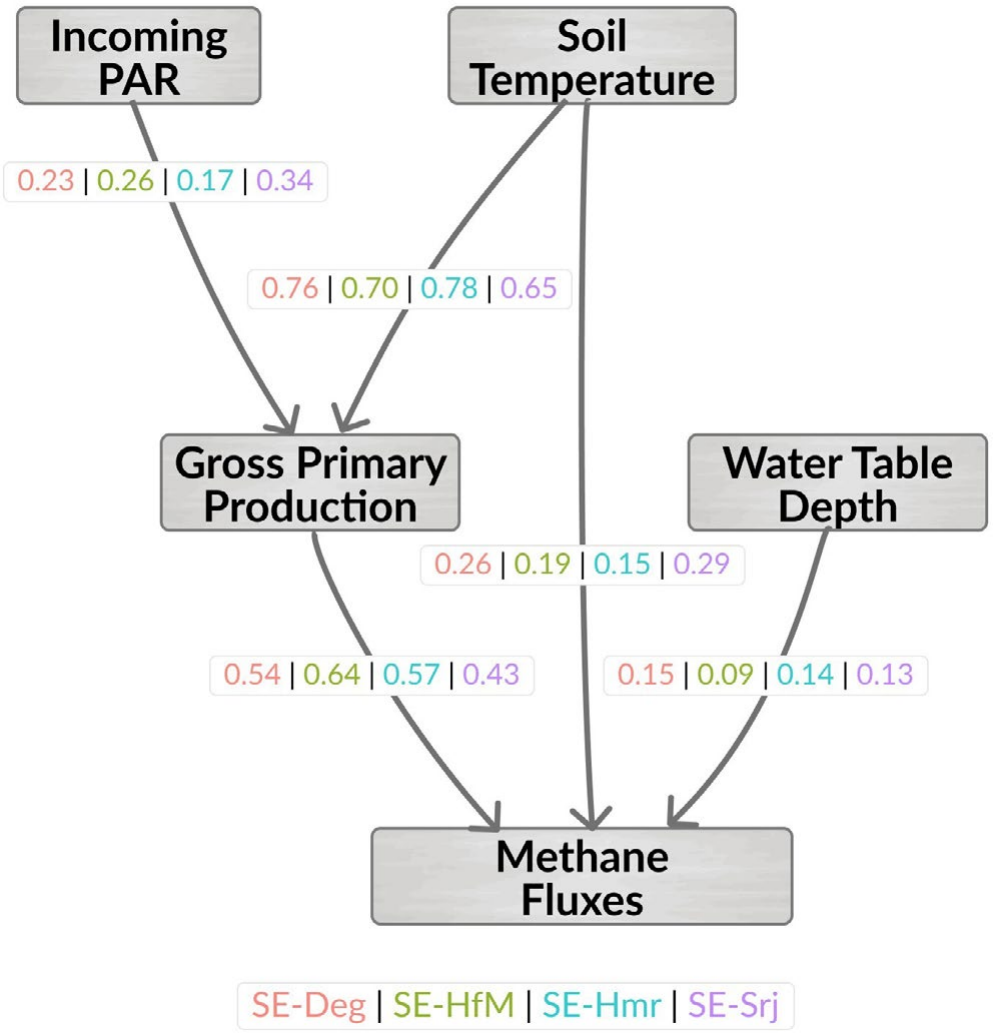
Path diagram showing the paths and effect sizes of soil temperature, incoming PAR, gross primary production, and water table depth in explaining daily methane fluxes. Colors correspond to site names (SE‐Deg, SE‐HfM, SE‐Hmr, and SE‐Srj) shown in the legend. All effect sizes were significant (*p* < 0.01) at all sites.

Path analysis performed based on results of the commonality analysis provided additional insights. The Comparative Fit Index (CFI), which integrates all multiple linear regressions in the path analysis (two regressions in this case, with GPP and FCH_4_ as dependent variables, respectively), ranged from 93%–98% (Table 5). SE‐Deg and SE‐HfM exhibited slightly higher CFI values (> 95%), compared to SE‐Hmr and SE‐Srj (CFI = 93%), in connection to a higher Root Mean Square Error of Approximation (RMSEA) at the latter two sites. *R* ^2^ was comparable (0.80–0.86) across sites for the first multiple linear regression, where PARin and Ts explained GPP (Table 5). The effect sizes of PARin (0.17–0.34) are smaller than that of Ts (0.65–0.78) on GPP across all sites (Figure 8). The WTD effect size in explaining daily FCH_4_ was consistently low (< 0.15) across all sites. In comparison, GPP had the highest effect size (0.43–0.64 depending on the site) among all three explanatory variables (GPP, Ts, and WTD). No apparent patterns were noted when comparing the effect sizes of each variable among the four sites, which suggest a consistent hierarchy of drivers for the temporal variations of FCH_4_ across the boreal mire complex.

**TABLE 5:** Fit metrics from the path analysis, that is, the coefficient of determination (*R* ^2^), comparative fit index (CFI), root mean square error of approximation (RMSEA), and the standardized root mean squared residual (SRMR).

Fit metrics	SE‐Deg	SE‐HfM	SE‐Hmr	SE‐Srj
*R* ^2^ GPP	0.86	0.80	0.81	0.85
*R* ^2^ FCH_4_	0.78	0.74	0.65	0.60
CFI	0.98	0.96	0.93	0.93
RMSEA	0.28	0.42	0.54	0.55
SRMR	0.02	0.04	0.05	0.05

### 3.5 Annual FCH_4_ From KRI Mire Sites Compared to Other Northern Peatlands

**FIGURE 9:**
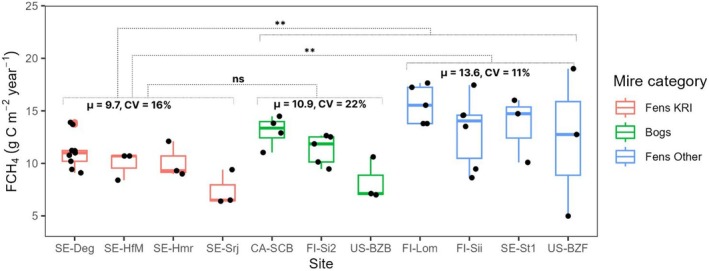
Annual sums of CH_4_ flux (FCH_4_) at the Kulbäcksliden Research Infrastructure (KRI) compared to data from eddy covariance Fluxnet‐CH_4_ sites (Table 2) split by mire type (Bog vs. Fen). Dot markers represent the annual FCH_4_ for each individual year. The boxplots show the first quartile (Q1), the median (Q2) and the third quartile (Q3) of the available FCH_4_ data per site, with boxplot colours representing the different mire categories (bogs, fens of the KRI, other fens). The whiskers stop at 1.5 × (Q3–Q1). Site codes on the *x*‐axis indicate country (SE = Sweden, FI = Finland, CA = Canada, US = United States) followed by site identifier as detailed in Table 2. The mean (μ) and coefficient of variation (CV) values shown for each mire category represent the average annual FCH_4_ and its variability across sites within that category. Statistical significance from Kruskal–Wallis tests between mire categories is indicated by: ‘ns’ (non‐significant, *p* > 0.05), and ‘**’ (highly significant, *p* < 0.01).

The statistical analysis did not reveal any significant difference of FCH_4_ at the four KRI fen complex sites and three distinct boreal bogs (CA‐SCB, Fi‐Si2, and US‐BZB) (Figure 9). However, a statistically significant difference (*p* < 0.01) existed between the mean annual FCH_4_ of the KRI fen complex sites and that of four other fen systems (and the combined data of all bogs and other fens). The similar intra‐group spatial variation in annual FCH_4_ represented by the coefficients of variation at the KRI fen sites (16%), the three bogs (22%) and the four other fen sites (11%) suggested that the spatial variability across the KRI mire complex is comparable to that exhibited among different mire systems across the circumboreal biome.

## 4 Discussion

### 4.1 High Variability of Ecosystem FCH_4_ Across a Boreal Mire Complex

The KRI with its replicated flux tower sites provided a unique opportunity to explore the variability of ecosystem‐scale FCH_4_ and environmental variables across a typical boreal mire complex over a three‐year period (2020–2022). Our results revealed that despite similar environmental conditions, substantial differences in the amplitude of daily FCH_4_ occurred across the four studied mire sites. Specifically, we showed that the variability in FCH_4_ across our fen complex is of similar magnitude compared to the variability across three different bog systems and four different fen sites within the circumboreal region. Our study, therefore, highlights an additional dimension of spatial variation that needs to be accounted for in the upscaling of single site flux measurements to the landscape and regional scales.

Although previous studies based on EC measurements have investigated the variability in FCH_4_ among different mire systems across the boreal biome (Knox et al. 2019), at present, there is a lack of empirical evidence of variations in FCH_4_ at the mesoscale across a mire complex. On the other hand, numerous studies based on chamber measurements reported significant spatial variability of FCH_4_ at the local plot scale within individual mire sites (Granberg et al. 1997; Lai et al. 2014; Ström et al. 2015; Svensson et al. 1999). These studies highlight that FCH_4_ is strongly regulated by the local microform characteristics, especially WTD, with higher emissions typically associated with lawns and hollows/pools, and lower emissions typically occurring at hummocks (Bubier et al. 1993; Granberg et al. 1997). In addition, the presence and composition of vascular plants at each of the microforms may further modify FCH_4_ (Öquist and Svensson 2002; Stewart et al. 2024). Furthermore, different mire types (e.g., fens/bogs, nutrient poor/nutrient rich) may co‐exist within the same mire complex, which may cause differences in ecosystem FCH_4_ given their distinct ecosystem properties and biogeochemical functioning (Euskirchen et al. 2024; Lindsay 2016; Zhang et al. 2021). This is particularly common in larger catchments and older mires with a more complex shape and surrounding topography, thus receiving differing nutrient and water inflow from their surroundings (Ehnvall, Ågren, et al. 2023). Altogether, this creates a potential for highly variable FCH_4_ at the mesoscale across a mire complex. Thus, a detailed understanding of the within‐mire complex variations, extending beyond simple mire type distinctions, is required to improve the upscaling of FCH_4_ from the site to the landscape scale.

### 4.2 Peat Decomposition Stage as a Key Indicator for the Spatial Variability of FCH_4_ Across a Mire Complex

The positive correlation between annual FCH_4_ and C:N ratio at the four mire sites (Figure 5) highlights that site characteristics such as nutrient status are important when studying the spatial variability of FCH_4_ in mires (Luan et al. 2019). While we did not measure nutrient availability in this study, C:N ratio can be considered a proxy, alongside bulk density, which gives an indication of the decomposition stage of a peatland (Krüger et al. 2015; Kuhry and Vitt 1996; Leifeld et al. 2020) and thereby provides insight on the nutrient quality and availability for methanogenesis. In fact, peat C:N ratio typically decreases as decomposition proceeds because C is preferentially lost with microbial activity (Biester et al. 2014; Watmough et al. 2022) while N is retained in most boreal mires, which are N‐limited systems. The lower C:N ratio and higher bulk density associated with a lower mire/catchment ratio (Figure 5; Figure S9) at the site SE‐Srj suggest more nutrient inflow from the relatively larger upland mineral area, which may have accelerated peat decomposition. This agrees with the theory that C accumulation in northern peatlands is limited by the strength of groundwater influence (and associated input of elements, nutrients and compounds), relative to rainwater influence, by stimulating decomposition (Mäkilä et al. 2001; Morris and Waddington 2011). In turn, more decomposed peat provides a poorer substrate for methanogenesis due to its lower content of labile carbon compounds and predominance of recalcitrant organic matter less accessible to methanogens (Hornibrook et al. 1997), possibly contributing to the lowest FCH_4_ being observed at the SE‐Srj site. The C:N ratio of 34 ± 3 to 43 ± 6 averaged over the 0–50 cm depth observed at the four mire sites is consistent with a previous study at the SE‐Deg site that determined a C:N ratio of 46 based on a peat core collected in 2009 (Larsson et al. 2017). These values are also consistent with the observed decrease of C:N ratio from 42 to 26 from surface peat to depths below 50 cm in Ontario, Canada (Wang et al. 2014). Northern peatlands exhibit a high variability in the C:N ratio, averaging 55 ± 33 and ranging from 34 ± 22 in Eastern Russia and Asia to 58 ± 31 in Fennoscandia (Loisel et al. 2014). This suggests a considerable potential for the C:N ratio to drive the variability in FCH_4_ across boreal mires sites.

In addition to the C:N ratio, the observed negative relationship between FCH_4_ and bulk density further supports the link between a decreased FCH_4_ and an increased degree of decomposition, since bulk density increases with peat decomposition (Boelter 1969). Apart from its effect on substrate quality, the collapse of pore space in the more degraded peat (Kleimeier et al. 2017) may also lead to reduced diffusion and ebullition (Baird et al. 2004), which may further explain the lower FCH_4_ observed at SE‐Srj. Thus, our results suggest that the effects of nutrient status and bulk density and their interaction with peat decomposition stage play a key role in regulating FCH_4_ across the mire complex.

In comparison to the important role of the decomposition stage in regulating FCH_4_ across the mire complex, the impact of other environmental variables remained limited. The close proximity of our four sites accounted for the similarities in meteorological variables (Ta and PAR). However, the small differences observed in WTD and Ts could be attributed to unique site characteristics such as differences in water inflow from the varying surrounding upland topography and in surface albedo, respectively. Nevertheless, these minor differences in WTD and Ts seemed insufficient to account individually for the spatial FCH_4_ variations observed across the mire complex.

Despite the known importance of vascular plants in regulating mire CH_4_ emissions (Girkin et al. 2020; Öquist and Svensson 2002; Yuan et al. 2024), our observations revealed lower FCH_4_ at sites with higher sedge AGB. This challenges the idea that plant‐mediated CH_4_ transport and substrate supply may play a key role in explaining spatial within‐mire complex variations in CH_4_ emissions. Possible explanations might include counterbalancing effects from increased oxygen transport via sedges that supports CH_4_ oxidation in deeper peat layers (Girkin et al. 2020; Määttä and Malhotra 2024; Turner et al. 2020) or that CH_4_ production is limited by other factors (e.g., decomposition rates) at these sites. It is further noteworthy that while the significant correlation between FCH_4_ and NDVI may suggest a vegetation‐related driver of the spatial variability in FCH_4_, the lack of correlation between mean NDVI with annual GPP and AGB reveals that NDVI in fact did not represent differences in plant productivity and biomass in these peatland ecosystems. Instead, NDVI was likely modified by other site characteristics such as differences in surface moisture, peat C:N ratio and/or the reflectance of different *Sphagnum* moss species (Bubier et al. 1997). In addition, the fact that the highest GPP (observed at SE‐Hmr) did not translate into the highest FCH_4_ (observed at SE‐Deg) further suggests that fresh substrate availability alone may not explain the spatial variability of FCH_4_ across a mire complex.

The observed CH_4_ concentrations in the peat matrix (Figure 4) in general mirrored the order in the magnitude of daily and frost‐free season sums of FCH_4_ among the four sites. Specifically, the lower CH_4_ concentrations observed at SE‐Srj corroborate the lower FCH_4_ observed from this site. It remains, however, unclear whether the lower CH_4_ concentrations were due to reduced CH_4_ production, higher CH_4_ oxidation, or a combination of both.

### 4.3 Soil Temperature and Plant Productivity as Key Drivers of the Temporal Variation in FCH_4_

Results from our path analysis revealed a consistent hierarchy of abiotic and biotic drivers for the temporal variations in FCH_4_ across the mire complex. This replicated evidence provides strong support for the identified primary drivers and further suggests that the variations in biogeophysical properties were within a limit that did not allow for a shift in the dominant controls across this mire complex. The highest first‐order effect of GPP in regulating the temporal variations in FCH_4_ at all sites highlights the important role of substrate availability (Bergman et al. 1998, 2000; Yuan et al. 2024), vegetation composition (Granberg et al. 2001; Riutta et al. 2020) and phenology (Ge et al. 2023; Whiting and Chanton 1993) in controlling the production and transport of CH_4_ to the atmosphere. The high second‐order GPP‐mediated Ts effect and low unique effects of individual variables (i.e., GPP, Ts and WTD) on FCH_4_ suggest that these variables jointly regulate FCH_4_ rather than one of them acting as a single dominant control. The joint driving effect of GPP and Ts could explain the elevated FCH_4_ summer peaks at SE‐Deg, given that the time series of both variables had the most robust and continuous daily‐scale coherences with the FCH_4_ time series throughout the summer period.

Temperature is one of the long‐known drivers of FCH_4_ as it regulates the microbial activity of methanogens (Bergman et al. 1998, 2000; Chang et al. 2021; Granberg et al. 2001; Westermann 1993) and methanotrophs (van Winden et al. 2012), with the different temperature sensitivities affecting the net FCH_4_ (Granberg et al. 1997). In addition, temperature affects all syntrophic and fermenting microbial activities generating substrates for methanogenesis. In our study, Ts exerted a substantial effect on FCH_4_ as reflected by the high total main effects of Ts and its second highest effect sizes. This is in line with strong correlations between seasonal variations in FCH_4_ and changes in Ts as found in previous studies in boreal mires (Long et al. 2010). However, the more important joint GPP‐Ts effect observed in this study indicates that the Ts control is significantly modified by other factors and should not be considered in isolation for explaining FCH_4_ (Chang et al. 2021).

We did not observe a strong control of WTD on the temporal variations of FCH_4_, neither as individual nor as interaction with other variables. This aligns with earlier findings from EC‐based studies suggesting that temporal variations in WTD may not act as the dominant control of FCH_4_ in wetlands when seasonal WTD fluctuations are limited (Knox et al. 2019). Furthermore, the irregular seasonal fluctuations of WTD throughout the growing season, combined with the fact that the wettest conditions generally occur in spring and autumn when temperatures are lower, may lead to confounding effects, making it difficult to isolate the distinct impact of WTD on FCH_4_.

### 4.4 Implications for Upscaling and Modelling Studies

Landscape settings, such as catchment size, composition, and age, play a significant role in shaping the heterogeneity of mire complexes (Ehnvall, Ågren, et al. 2023). For example, larger catchments may encompass a wider range of microhabitats, leading to differing nutrient availability across the mire complex. Additionally, the larger the proportion of mire relative to the mineral soil in a catchment, the more difficult it will be for nutrients to reach different parts of the mire complex. Older mires, having undergone more extensive ecological succession and peat accumulation, may exhibit a more complex structure, resulting in greater variability in nutrient status and peat decomposition stage. These landscape factors can collectively interact with other environmental variables like temperature and nutrient availability, adding complexity to the relationship between mire complex characteristics and FCH_4_. Thus, regional peatland FCH_4_ studies ought to resolve the mesoscale heterogeneities at mire complexes before upscaling to the landscape scale to reduce biases in the global carbon budget.

The accuracy of existing CH_4_ biogeochemistry models is only as good as the representativeness of the data used to develop them (Bridgham et al. 2013). The considerable variation in cumulated annual FCH_4_ observed between the four mires within a single mire complex in this study implies that peatland ecosystem models may inaccurately estimate FCH_4_ at the regional to biome scales when assuming a binary classification of peatland types, that is, fen versus bog. Although our study focused on a single mire complex, the observed variability in FCH_4_ underscores the importance of representativeness in the data used for calibrating global FCH_4_ models. Based on our findings, we suggest that these models should be capable of capturing the differences in peat decomposition stage and nutrient status to accurately simulate the spatial variations of FCH_4_ across large mire complexes and different mire types.

## Author Contributions

**Koffi Dodji Noumonvi:** conceptualization, data curation, formal analysis, methodology, software, validation, visualization, writing – original draft. **Mats B. Nilsson:** conceptualization, funding acquisition, investigation, methodology, project administration, resources, supervision, validation, writing – review and editing. **Joshua L. Ratcliffe:** conceptualization, data curation, investigation, methodology, supervision, validation, writing – review and editing. **Mats G. Öquist:** conceptualization, investigation, methodology, resources, supervision, writing – review and editing. **Natascha Kljun:** supervision, writing – review and editing. **Johan E. S. Fransson:** supervision, writing – review and editing. **Järvi Järveoja:** resources, writing – review and editing. **Anders Lindroth:** conceptualization, resources, writing – review and editing. **Gillian Simpson:** data curation, writing – review and editing. **Jacob Smeds:** data curation, writing – review and editing. **Matthias Peichl:** conceptualization, data curation, funding acquisition, investigation, methodology, project administration, resources, supervision, validation, writing – review and editing.

## Conflicts of Interest

The authors declare no conflicts of interest.

## Supporting information


Data S1.

## Data Availability

The data that support the findings of this study are openly available in Dryad at https://doi.org/10.5061/dryad.k0p2ngfk4. Methane flux data was obtained from Fluxnet‐CH4 V2.0 at https://doi.org/10.18140/FLX/1669613 (Scotty Creek Bog in Canada), https://doi.org/10.18140/FLX/1669668 (Bonanza Creek Thermokarst Bog), https://doi.org/10.18140/FLX/1669669 (Bonanza Creek Rich Fen), https://doi.org/10.18140/FLX/1669660 (Stordalen grassland), https://doi.org/10.18140/FLX/1669638 (Lompolojankka), https://doi.org/10.18140/FLX/1669659 (Degero), https://doi.org/10.18140/FLX/1669639 (Siikaneva‐2 Bog), and https://doi.org/10.18140/FLX/1669640 (Siikaneva).
