# Supplementary material for: Variations in Ecosystem‐Scale Methane Fluxes Across a Boreal Mire Complex Assessed by a Network of Flux Towers
Source: Glob Chang Biol. 2025 May 5;31(5):e70223. doi: 10.1111/gcb.70223 (PMC12051366; doi:10.1111/gcb.70223)
Supplement: Supplementary file 1 — Data S1. [file GCB-31-e70223-s001.pdf]

SUPPLEMENTARY INFORMATION FOR THE ARTICLE:

Variations in ecosystem-scale methane fluxes across a boreal mire complex assessed by a network of flux towers

[1]Noumonvi Koffi Dodji, [1]Mats B. Nilsson, [1,2]Joshua L. Ratcliffe, [1]Mats G. Öquist, [3]Natascha Kljun, [4]Johan E. S. Fransson, [1]Järvi Järveoja, [5]Anders Lindroth, [1]Gillian Simpson, [1]Jacob Smeds, [1]Matthias Peichl

[1] Department of Forest Ecology and Management, Swedish University of Agricultural Sciences, 901 83 Umeå, Sweden
[2] Unit for Field-Based Forest Research, Swedish University of Agricultural Sciences, 922 91 Vindeln, Sweden
[3] Centre for Environmental and Climate Science, Lund University, 223 62 Lund, Sweden
[4] Department of Forestry and Wood Technology, Linnaeus University, 351 95 Växjö, Sweden
[5] Biodiversity and Ecosystem services in a Changing Climate, Lund University, 223 62 Lund, Sweden

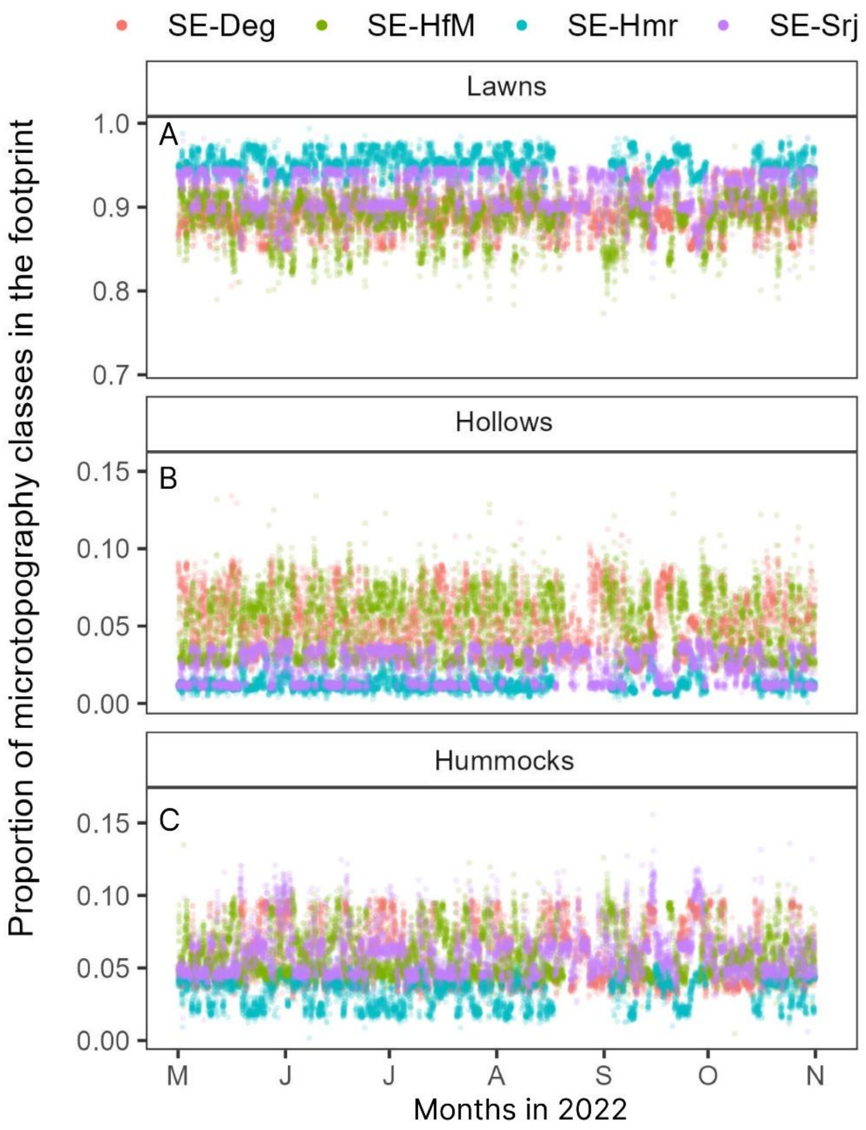

Figure S1. Extracted half-hourly footprint-weighted proportion of the three microtopography classes, lawns (A), hollows (B) and hummocks (C) at each of the four sites, SE-Deg, SE-HfM, SE-Hmr and SE-Srj represented by the different colors. Shown are data from May to October of the year 2022. The microtopography was mapped using using the HuHoLa model (Noumonvi et al., 2025 - in review), and the half-hourly footprint was calculated using the two-dimensional flux footprint prediction model (Kljun et al., 2015).

Table S1: Instrumentation used for measuring greenhouse gas fluxes and environmental variables at the study sites, Degerö Stormyr (SE-Deg), Hälsingfors Stormyran (SE-HfM), Hålmyran (SE-Hmr), Stortjärn (SE-Srj).

**Variable**	**SE-Deg**	**SE-HfM**	**SE-Hmr**	**SE-Srj**
**CO2, H2O**	LI-7200	-2020: EC 155 CS<br>-2021 - Now: Picarro G2311-f	Picarro G2311-f	Picarro G2311-f
**CH4**	LGR FGGA 911-0010	-2020: LI-7700<br>2021 - Now: Picarro G2311-f	Picarro G2311-f	Picarro G2311-f
**Wind speed and direction**	Gill HS-50	Metek uSonic-3 Class A	Metek uSonic-3 Class A	Metek uSonic-3 Class A
**Air temperature**	Rotronic MP102H-331000	HC2S3 CS	HC2S3 CS	HC2S3 CS
**Soil temperature**	-2020-2021: Micro-step Pt100 probe<br>-2022-Now: Fischer Pt100	TO3R TOJO Skogsteknik	TO3R TOJO Skogsteknik	TO3R TOJO Skogsteknik
**Water table depth**	CS450	CS451	CS451	CS451
**Precipitation**	-2020-2021: Geonor T200b<br>-2022-Now: Lambrecht Rain[e]H3	ARG100	ARG100	ARG100
**Solar radiation**	CNR4	NR01 Campbell Scientific	NR01 Campbell Scientific	NR01 Campbell Scientific
**Photosynthetically active radiation**	Li-190	Li-190	Li-190	Li-190

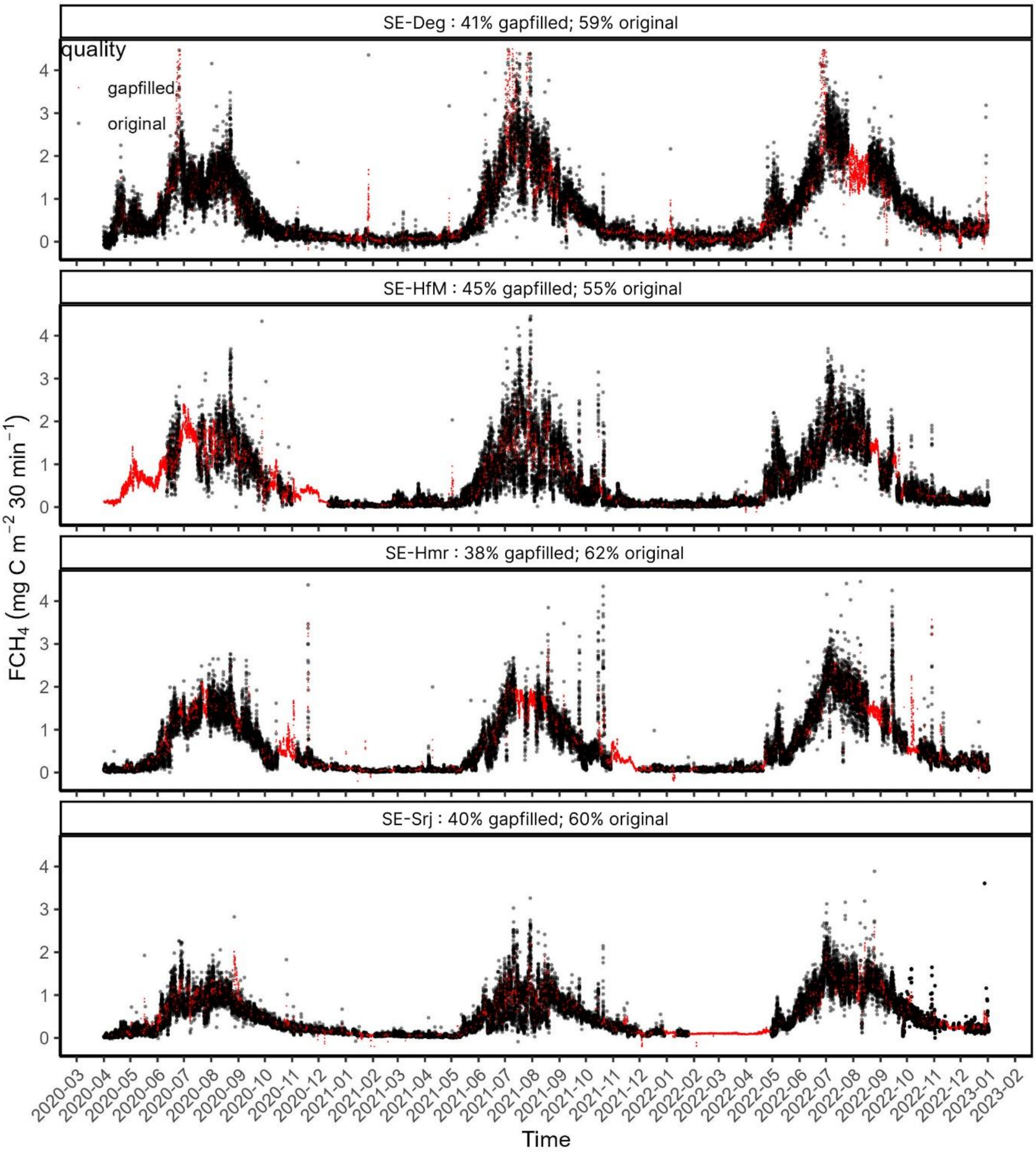

Figure S2. Half-hourly methane flux (FCH_4) time series. Black dots represent original data, and red dots are gapfilled data. Each panel is labelled with the corresponding site name. Data for year 2020 started in April 2020, and period before that was not treated as missing data in this calculation.

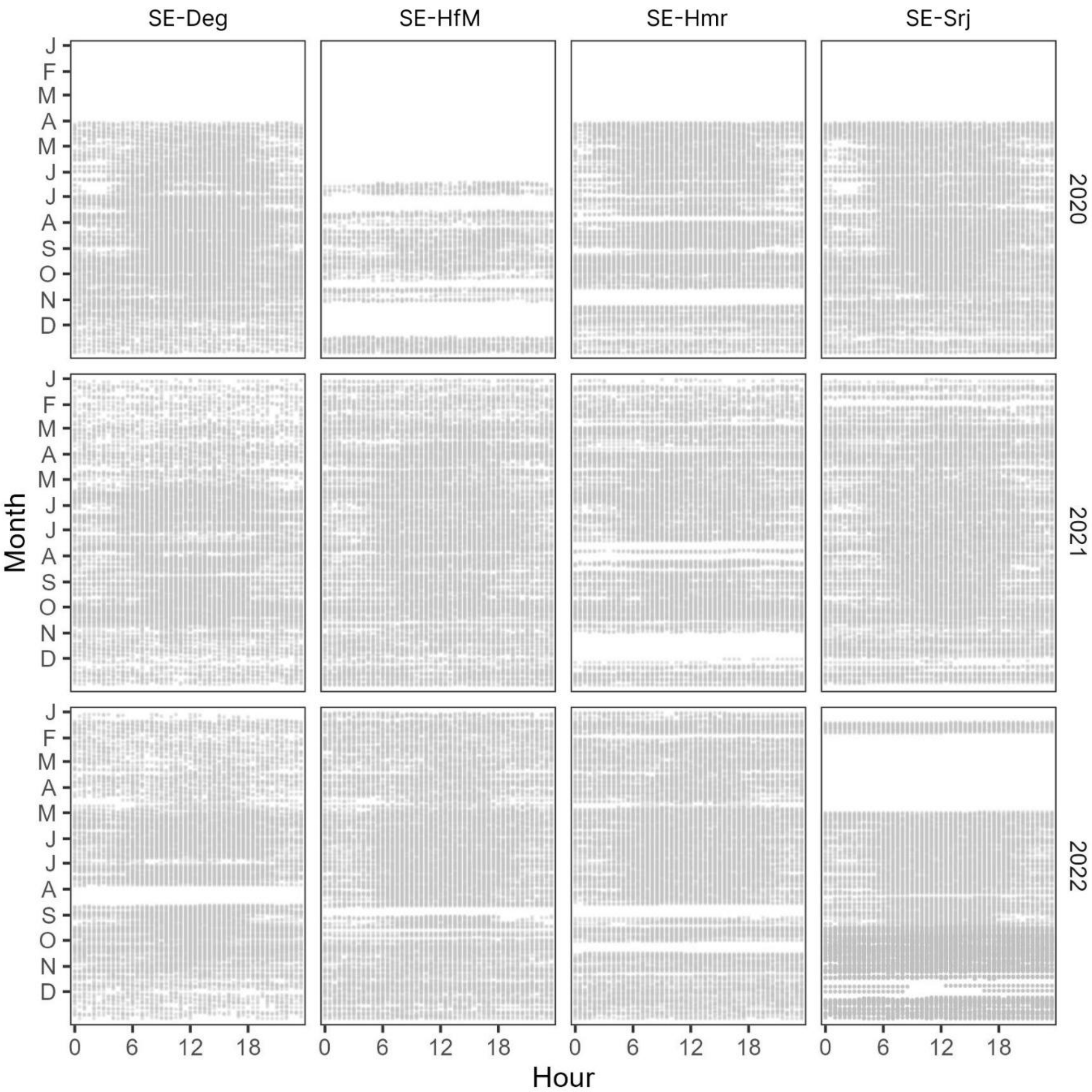

Figure S3. Half-hourly FCH_4 data available per year at each site. Gaps represent missing data after filtering, and before gapfilling.

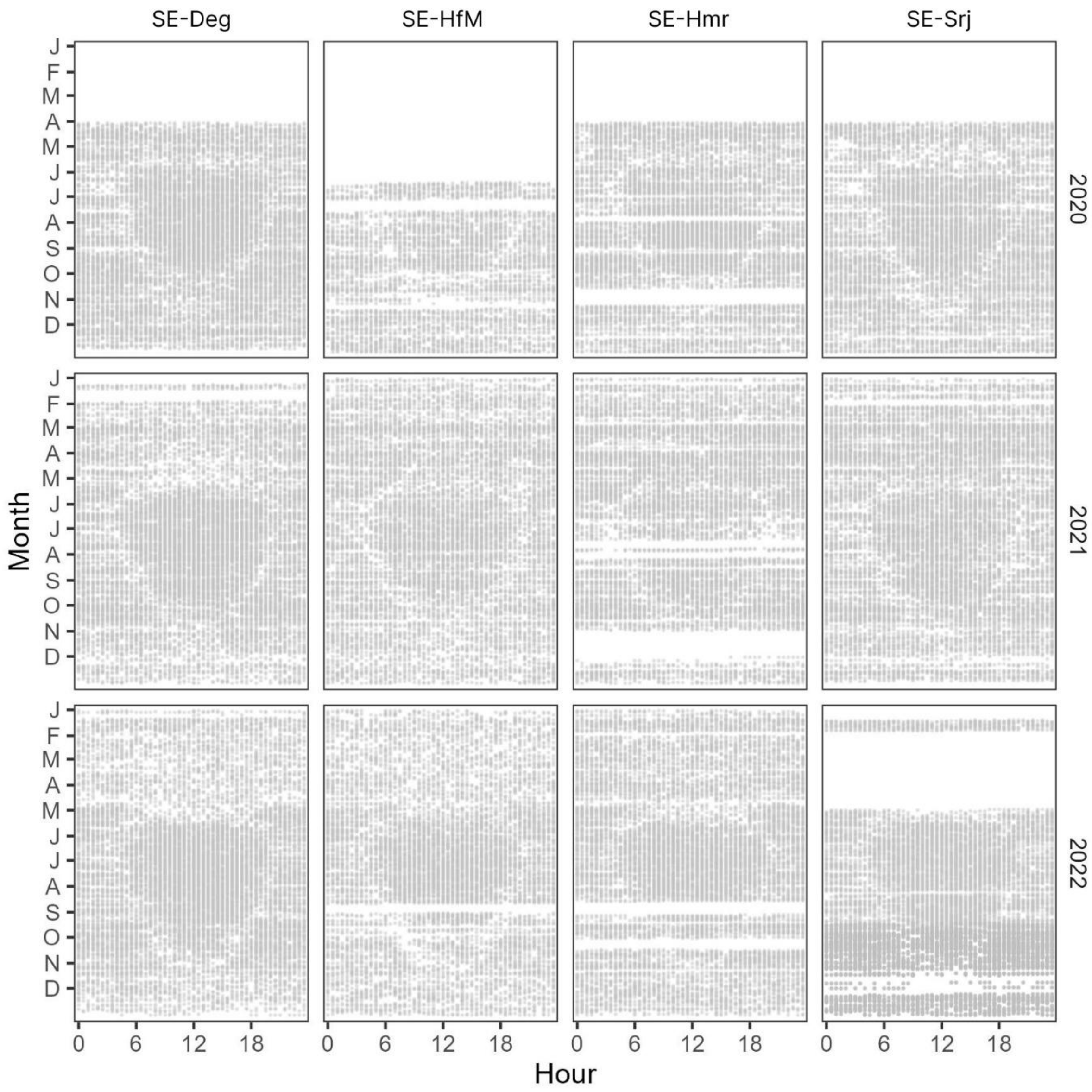

Figure S4. Half-hourly data of the Net Ecosystem Exchange (NEE) available per year at each site. Gaps represent missing data after filtering, and before gapfilling.

Table S2: Percentage of available data per year for NEE and CH_4 fluxes (FCH_4) at all sites. Data for year 2020 started in April 2020, and period before that was not treated as missing data in this calculation.

Frost-free Season	Site	NEE (%)			FCH_4 (%)		
		2020	2021	2022	2020	2021	2022
YES	SE-Deg	68	68	70	76	72	68
	SE-HfM	35	54	54	31	71	69
	SE-Hmr	53	49	56	62	61	65
	SE-Srj	60	59	66	72	72	76
NO	SE-Deg	58	37	36	54	38	41
	SE-HfM	25	45	37	18	60	64
	SE-Hmr	55	45	49	65	50	68
	SE-Srj	51	48	16	66	56	23

Table S3: Frost-free seasons during 2020-2022 at the four mire sites. Frost-free season is defined as the period during which soil temperature at 10 cm depth remains consistently above 1°C for at least five consecutive days.

Year	Site	start	end
2020	SE-Deg	2020-05-07	2020-12-06
	SE-HfM	2020-05-07	2020-12-02
	SE-Hmr	2020-05-09	2020-11-25
	SE-Srj	2020-05-22	2020-11-22
2021	SE-Deg	2021-05-07	2021-11-07
	SE-HfM	2021-05-11	2021-11-19
	SE-Hmr	2021-05-13	2021-11-13
	SE-Srj	2021-05-15	2021-11-08
2022	SE-Deg	2022-05-07	2022-11-15
	SE-HfM	2022-05-11	2022-11-17
	SE-Hmr	2022-05-07	2022-11-17
	SE-Srj	2022-05-19	2022-11-14

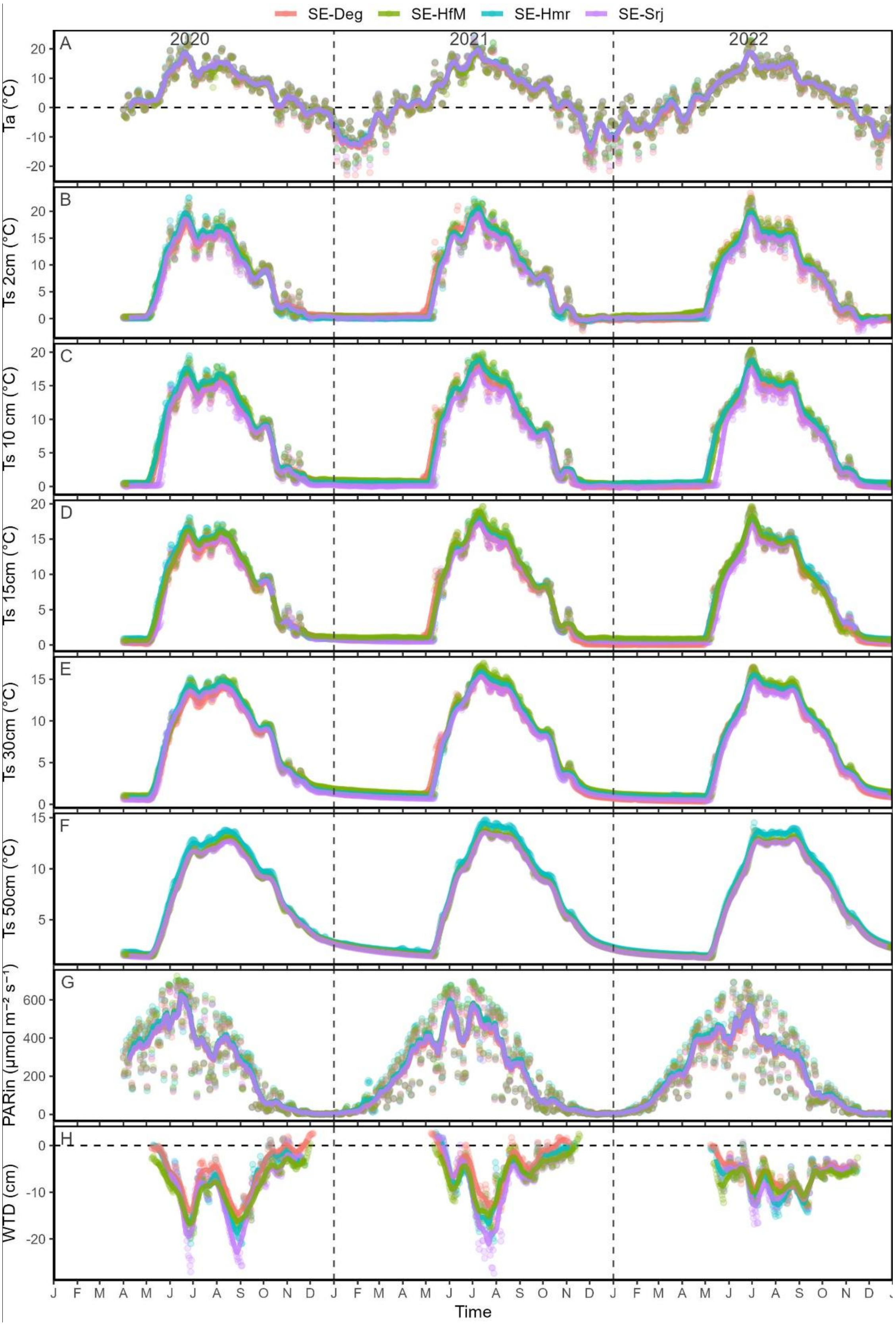

Figure S5. Half-hourly data for environmental variables, including (A) Air temperature at 2m height, (B) to (F) Soil temperature at 2, 10, 15, 30, and 50 cm depth, (G) incoming Photosynthetically Active Radiation, (H) Frost-free season water table depth.

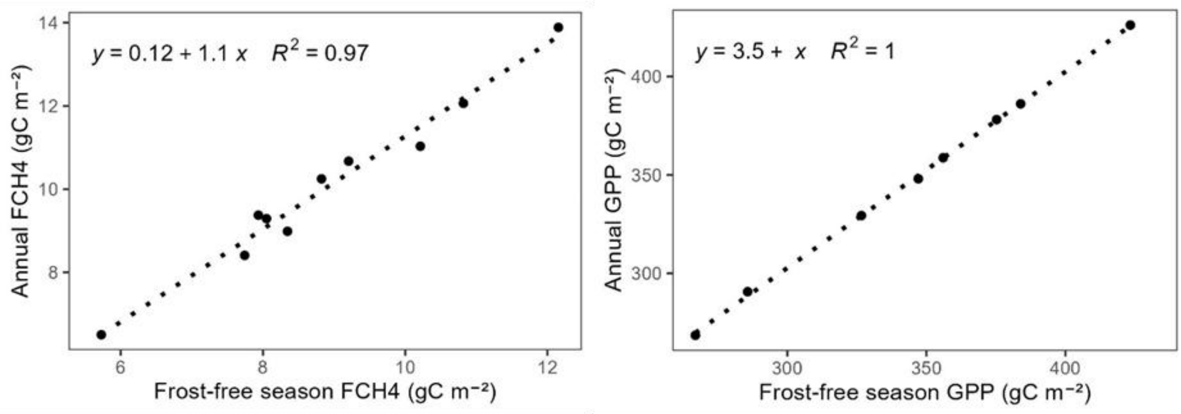

Figure S6. Relationship between annual and frost-free season fluxes, used for estimating annual fluxes for year 2020.

Table S4: Average of the three frost-free seasons and years of FCH_4 and GPP (± standard deviation).

	Frost-free season		Annual	
**Site**	**FCH_4**	**GPP**	**FCH_4**	**GPP**
SE-Deg	10.1 ± 2.1	340 ± 20	11.4 ± 2.3	342 ± 21
SE-HfM	8.9 ± 1.1	279 ± 11	10 ± 1.4	282 ± 12
SE-Hmr	9.1 ± 1.4	410 ± 31	10.2 ± 1.7	413 ± 30
SE-Srj	6.4 ± 1.3	354 ± 29	7.4 ± 1.7	356 ± 29

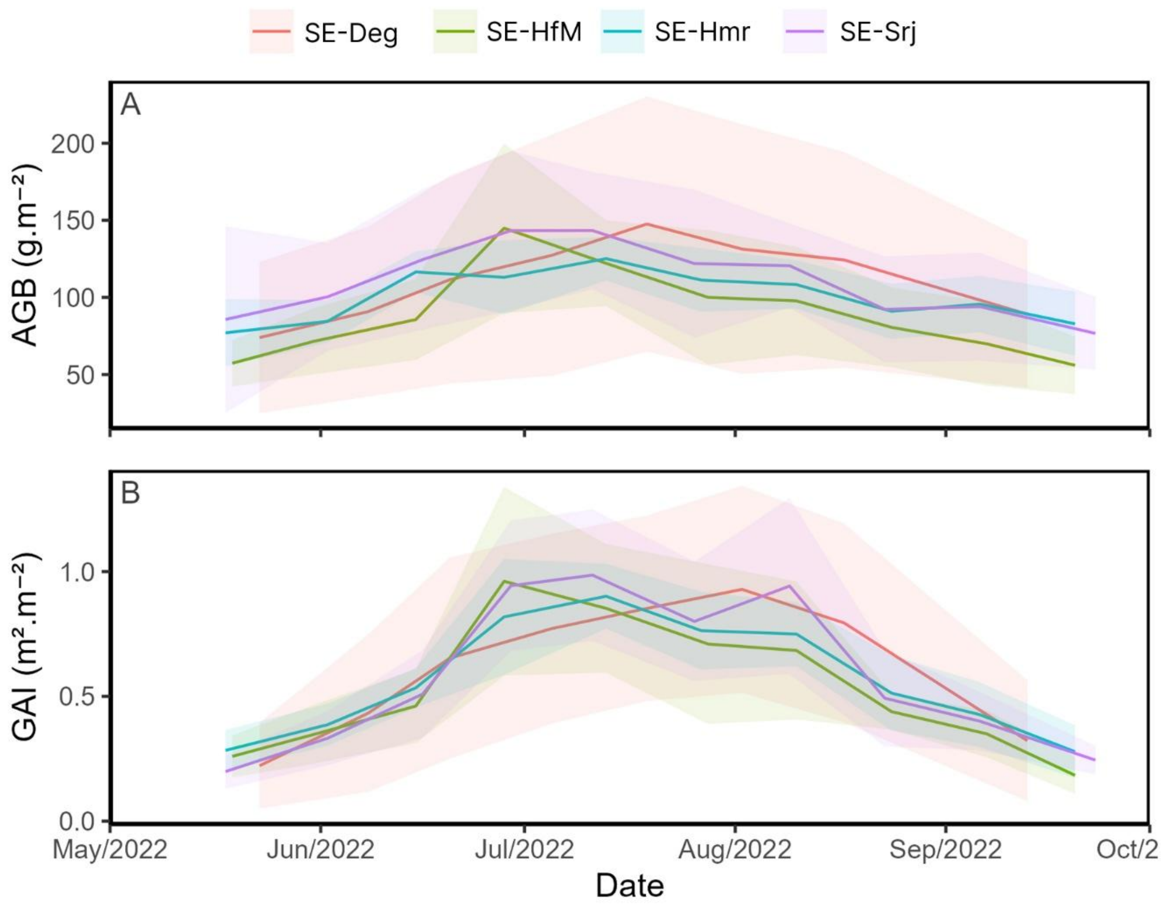

Figure S7. Above ground biomass (AGB) and green area index (GAI) at the four mire sites for year 2022.

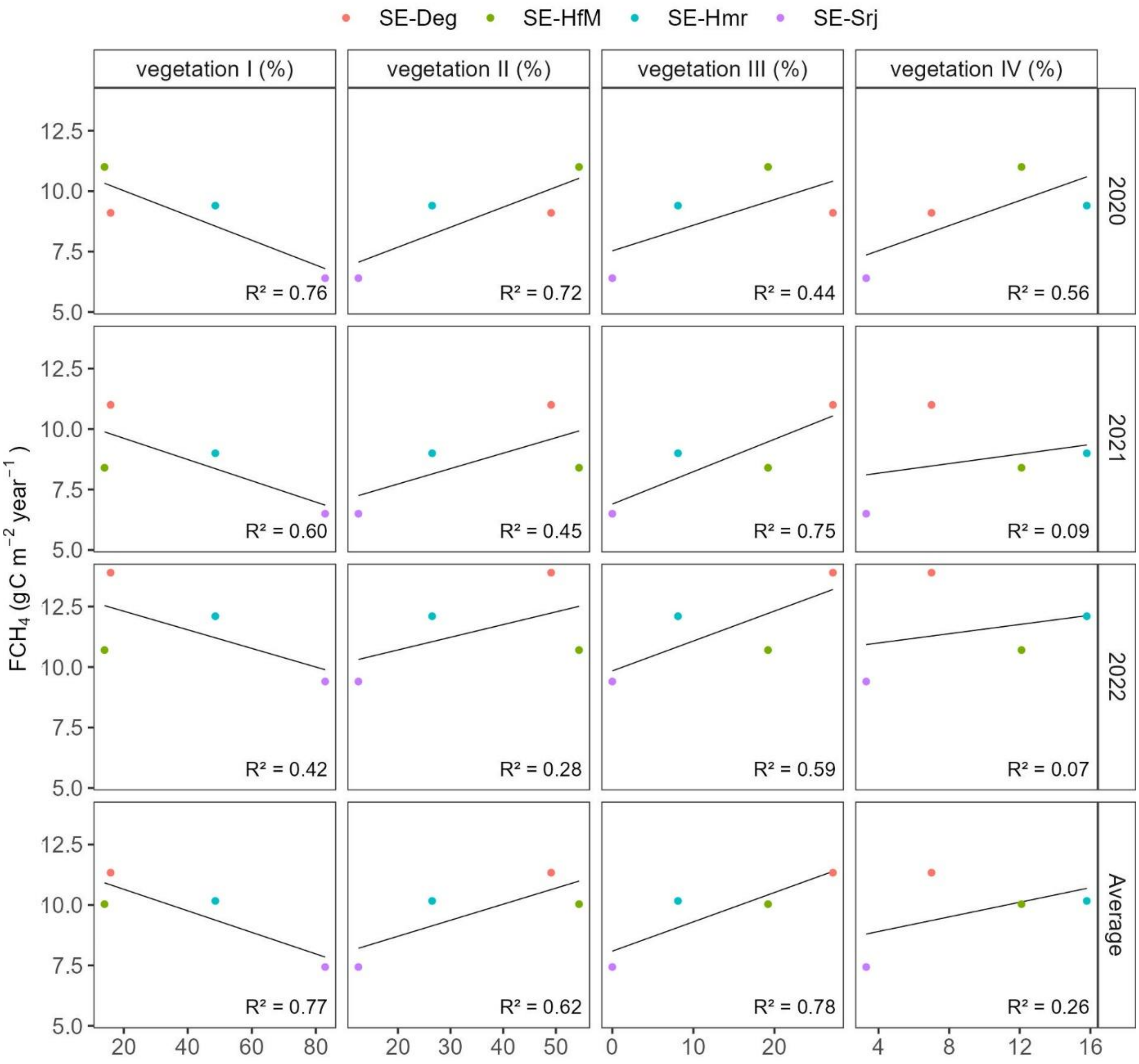

Figure S8. Correlations between annual and 3-year mean methane fluxes (FCH_4) with site vegetation characteristics, i.e. the four dominant vegetation classes in the 80% footprint of the eddy covariance measurements. None of the linear relationships was significant.

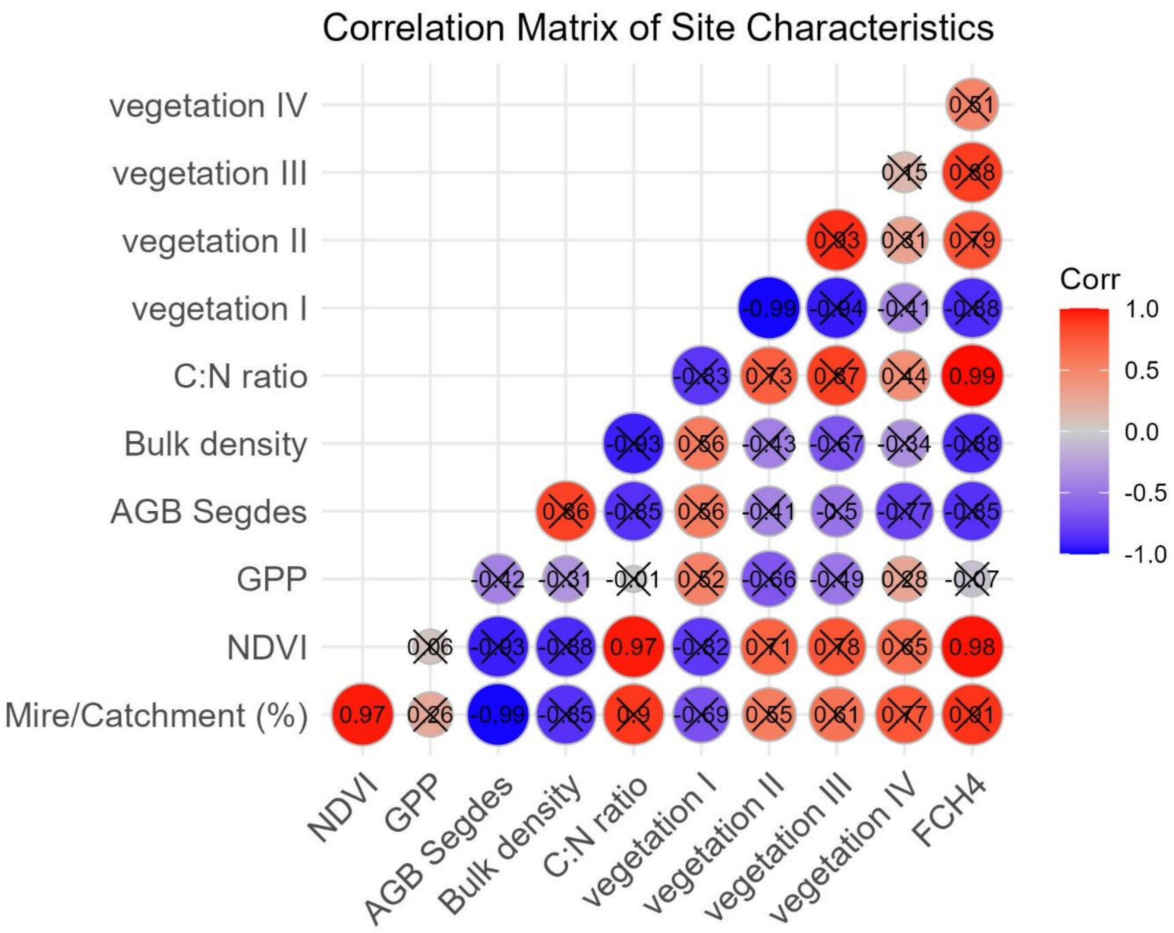

Figure S9. Pearson correlation plots between different annual site characteristics and methane fluxes (FCH_4). NDVI is the normalized difference vegetation index, GPP is the gross primary production, AGB is the above ground biomass, and vegetation I to IV represent different vegetation classes. The numbers in the circles indicate the Pearson's correlation coefficient between two variables, the blue correlation circles indicate negative correlation, while the red correlation circles indicate a positive correlation. The crossed correlation circles indicate non-significant correlations.

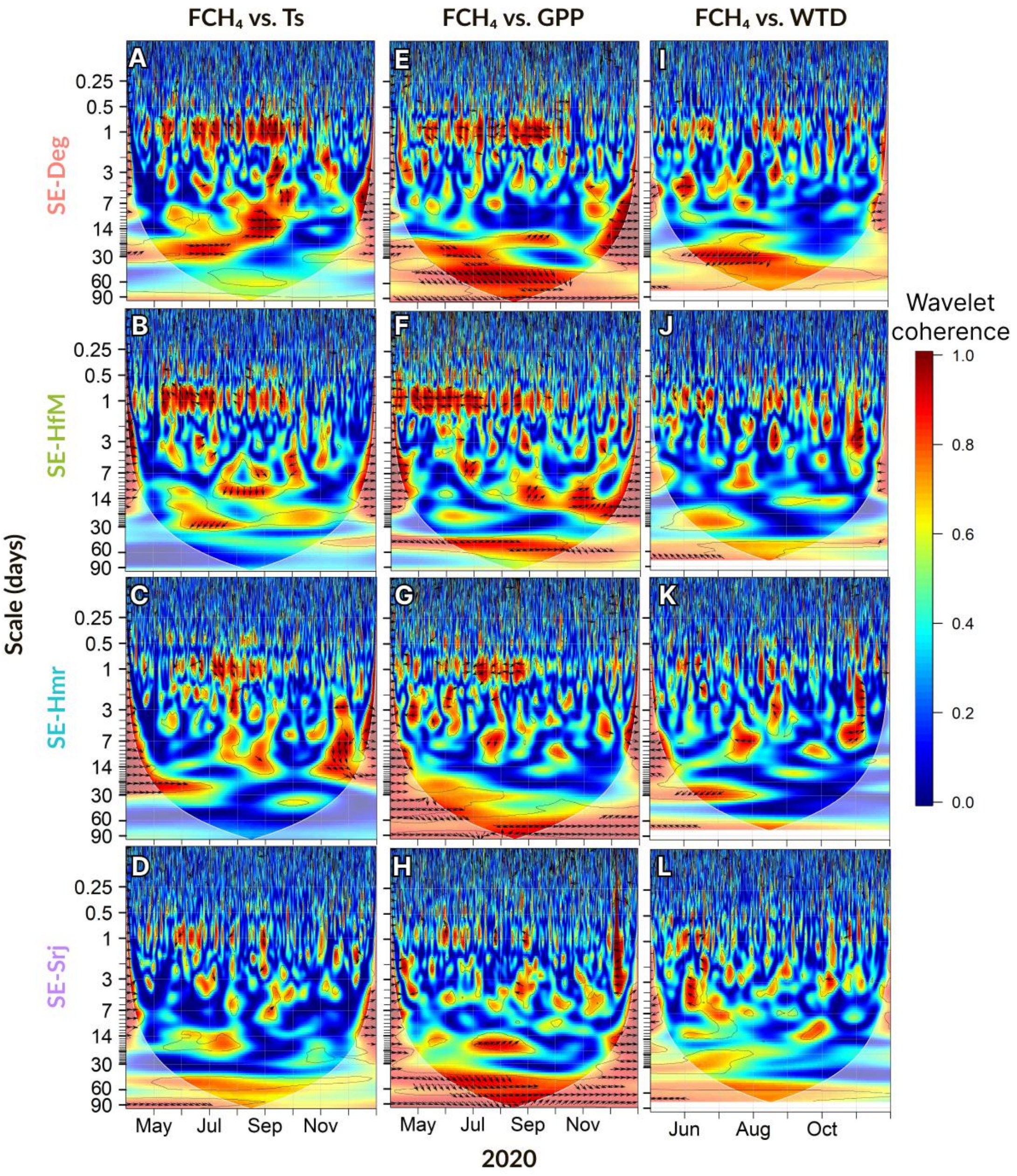

Figure S10. Wavelet coherence between half-hourly FCH_4 and Ts 10 cm (A-D), GPP (E-H), and WTD (I-L) in 2020, whith each row representing one of the four sites (SE-Deg, SE-HfM, SE-Hmr and SE-Srj). The greyed area is the area outside of the cone of influence, i.e. 5% significance level; the arrows indicate the phase between the two variables at very high coherence (red areas).

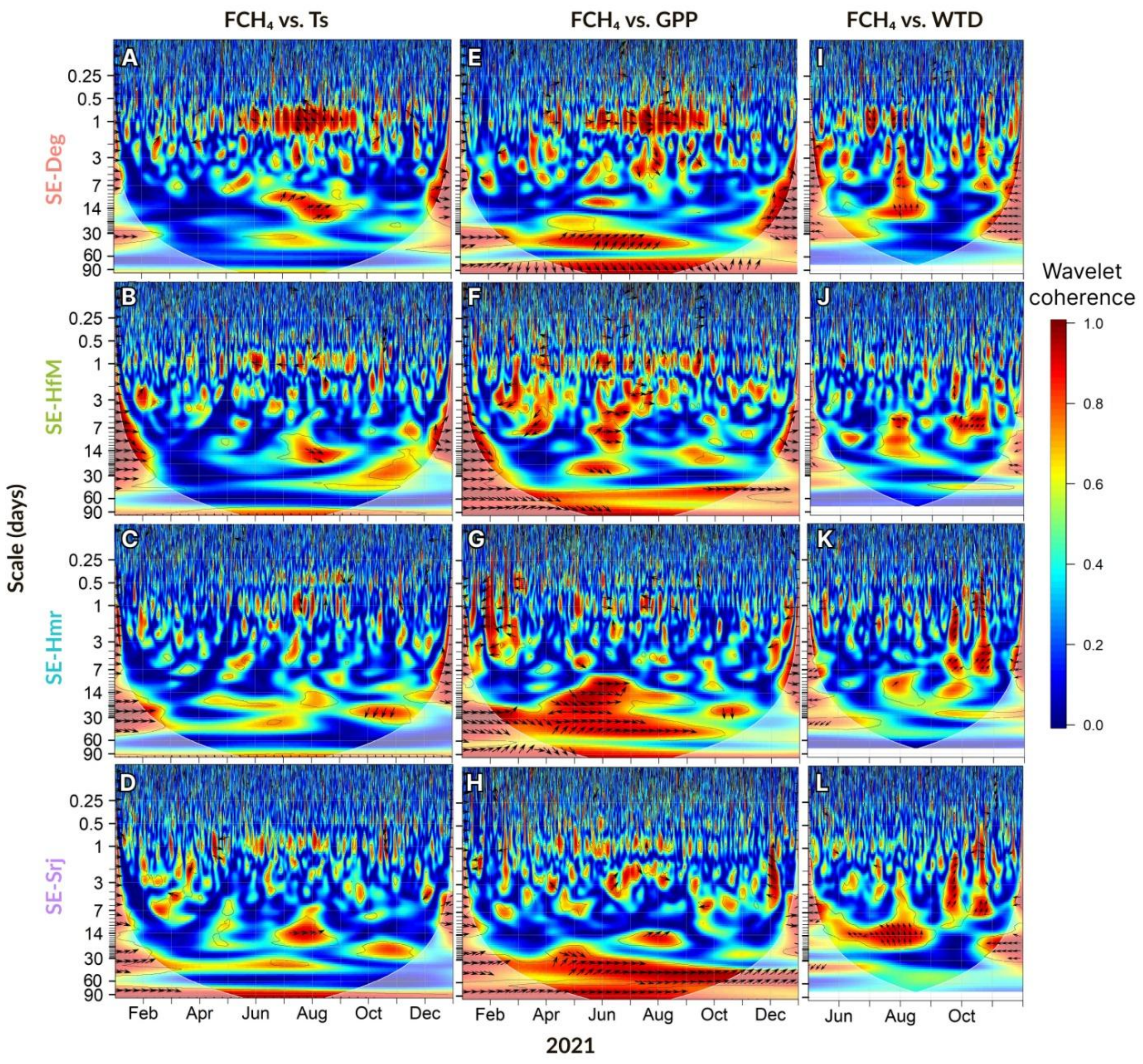

Figure S11. Wavelet coherence between half-hourly FCH$_4$ and Ts 10 cm (A-D), GPP (E-H), and WTD (I-L) in 2021, whith each row representing one of the four sites (SE-Deg, SE-HfM, SE-Hmr and SE-Srj). The greyed area is the area outside of the cone of influence, i.e. 5% significance level; the arrows indicate the phase between the two variables at very high coherence (red areas).

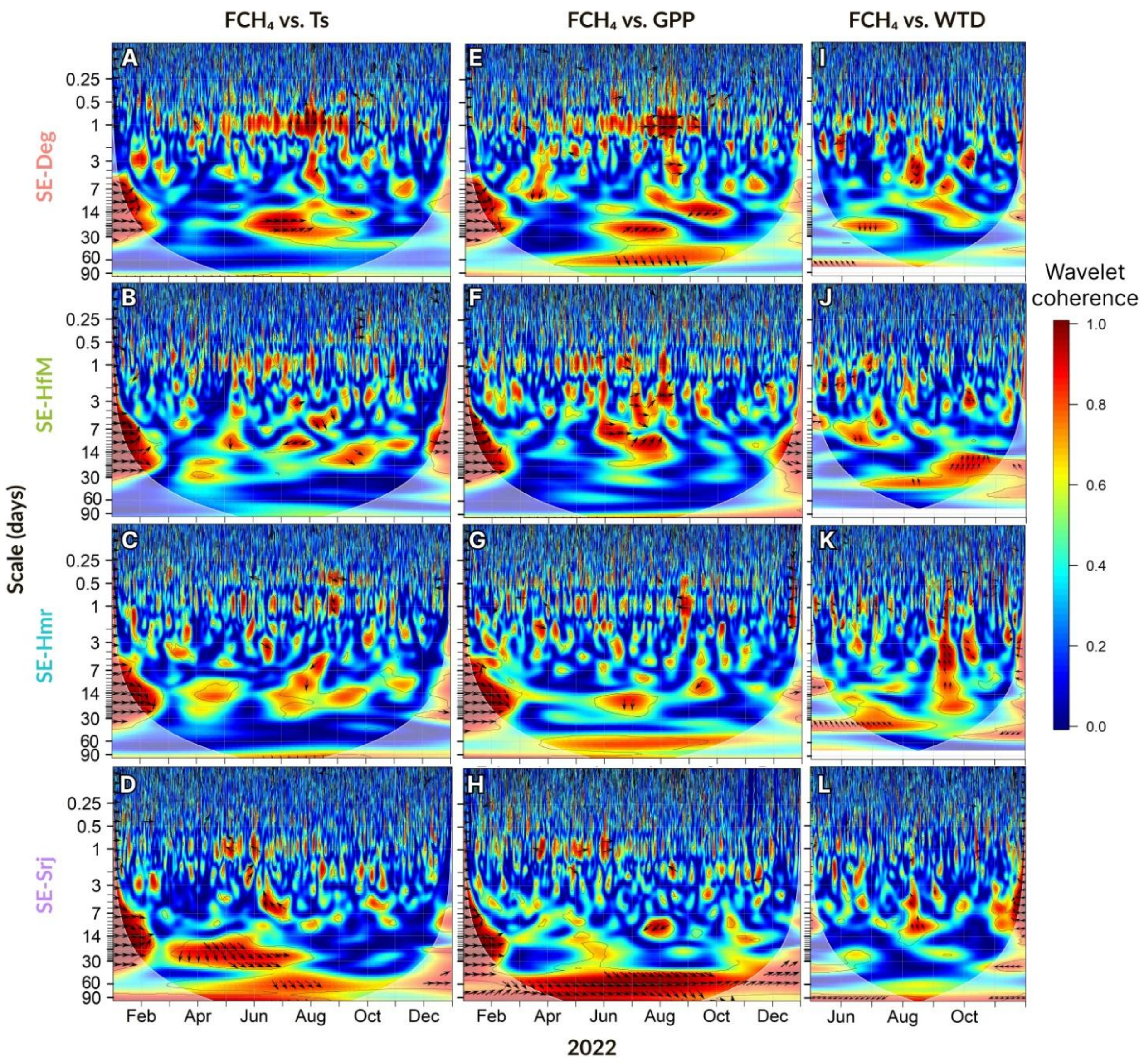

Figure S12. Wavelet coherence between half-hourly FCH_4 and Ts 10 cm (A-D), GPP (E-H), and WTD (I-L) in 2022, with each row representing one of the four sites (SE-Deg, SE-HfM, SE-Hmr and SE-Srj). The greyed area is the area outside of the cone of influence, i.e. 5% significance level; the arrows indicate the phase between the two variables at very high coherence (red areas).

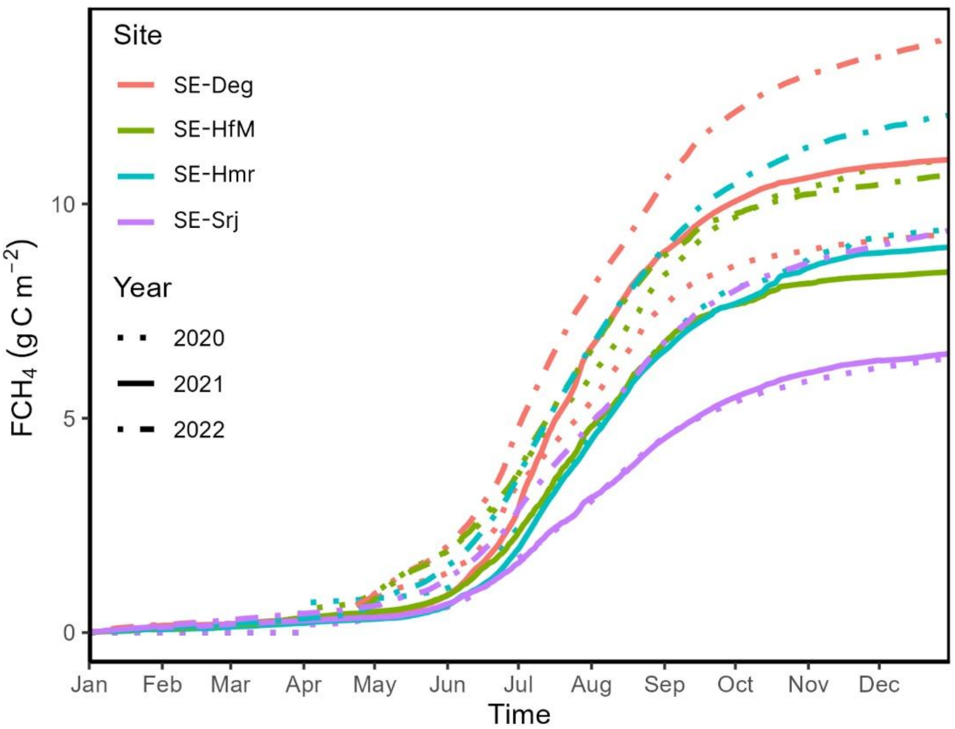

Figure S13. Cumulative FCH_4 plots. Measurements at SE-HfM, SE-Hmr and SE-Srj started in April 2020, and the starting values in April were estimated as the difference between annual fluxes (calculated from an established linear relationship between the frost-free season fluxes and annual fluxes from all sites together, supplementary materials Figure S5) and the sum of fluxes from April to the end of the year.

References

Kljun, N., Calanca, P., Rotach, M.W., Schmid, H.P., 2015. A simple two-dimensional parameterisation for Flux Footprint Prediction (FFP). Geosci. Model Dev. 8, 3695–3713. https://doi.org/10.5194/gmd-8-3695-2015

Noumonvi, K.D., Havertz, N.H., Bohlin, J., van der Linden, S., Nilsson, M.B., Peichl, M., 2025. HuHoLa: A novel Hummock-Hollow-Lawn mire microtopography modelling approach. (In review)
